# Lived experiences of Type 1 diabetes patients visiting a tertiary care hospital of Nepal: A descriptive phenomenological study

**DOI:** 10.1371/journal.pgph.0005810

**Published:** 2026-01-13

**Authors:** Anusa Parajuli Aryal, Bijay Thapa, Binod Regmi, Shital Bhandary

**Affiliations:** 1 School of Public Health, Patan Academy of Health Sciences, Lalitpur, Nepal; 2 National Institute of Arthritis and Musculoskeletal and Skin Disease, National Institutes of Health, Bethesda, Maryland, United States of America; Tribhuvan University, NEPAL

## Abstract

Type 1 diabetes is a non-preventable chronic disease that predominantly affects young people, accounting for 10–15% of all diabetes cases. The condition is multidimensional, affecting various aspects of life, and daily living requires consistent effort, lifestyle modification, and close monitoring. This study aimed to explore the lived experiences of patients with Type 1 diabetes attending the outpatient department of Patan Hospital, focusing on their perspectives on living with the disease condition. A descriptive phenomenological research design was used for the study. Data were collected from 22 purposively selected patients who attended the Medicine and Pediatrics Outpatient Department (OPD) of Patan Hospital. Patients aged 15 years and above, with at least one year since initial diagnosis, were included in the study. Colaizzi’s descriptive phenomenological method was used for data analysis. Ten themes emerged from the analysis: (1) Initial hospitalization (2) Solidarity in illness and support systems (3) Learning process and acceptance (4) Changes and adjustments (5) Diabetes management challenges (6) Concerns and worries (7) Socioeconomic and structural challenges (8) Healthcare experiences (9) Type 1 diabetes-related stigma (10) Experiences of living with Type 1 diabetes during the COVID-19 pandemic. The study highlights a lack of awareness in the community on Type 1 diabetes, experiences of stigma were also reported by the participants. It also emphasizes the need for regular parental counseling to prevent overprotection. Financial burden appeared as a significant challenge in the study. The study further suggests that authorities ensure access to insulin and other essential medical supplies during lockdowns, as participants reported difficulties in obtaining them. School-related difficulties point towards the importance of effective implementation of the “School Health Nurse” program. These findings indicate the need for greater awareness, psychological support, and healthcare preparedness to improve the quality of life of individuals living with Type 1 diabetes.

## Introduction

Type 1 diabetes is a non-preventable chronic disease with early onset, mostly in childhood, constituting about 10–15% of all diabetes cases worldwide [1]. It results from autoimmune destruction of the pancreatic beta cells, leading to insulin deficiency, thereby causing hyperglycemia [2]. Daily management of Type 1 diabetes requires following strict regimens, sustained lifestyle changes, and constant vigilance to maintain glycemic control [3–5]. Patients are subjected to lifelong blood glucose monitoring, insulin injections, and continual self-care, which may contribute to psychological distress. [6] Despite clinical guidance, gaps frequently exist between medical recommendations and actual patient practices [7]. Social stigma and reluctance to disclose the diabetic status add further challenges [8]. Families are also profoundly affected, and crises such as the COVID-19 pandemic exacerbate stress and vulnerability [9,10]. Qualitative studies, especially phenomenological approaches, have been widely used in various countries outside Nepal to study the detailed lived experiences of Type 1 diabetes patients [11–13]. In such studies, experiences related to the worries of an uncertain future, stigma related to disclosure, social support, and striving for autonomy were commonly shared by the participants [14,15]. Other studies have revealed that patients experience issues related to financial constraints, medication adherence, exercise, side effects of medications, and family relationships [16–23].

However, most of this evidence comes from Western contexts. Only a limited number of studies have been conducted in South Asia, and in Nepal, existing research has been predominantly quantitative, focusing on clinical or epidemiological aspects [24–26]. Therefore, a gap remains in understanding the subjective realities of Nepalese patients living with Type 1 diabetes. This qualitative study was designed to explore these lived experiences, identifying common elements and patterns that may inform patient-centered care in the Nepalese context.

## Materials and methods

**Study design:** A descriptive phenomenological research design was used in this study. Descriptive phenomenology captures the universal essence of a phenomenon through common elements of lived experiences shared by individuals with that experience [27]. This design focuses on rich descriptions of experiences, describing how the experiences are lived to communicate the essential structure of the phenomenon as experienced by the participants. It reduces the researcher’s bias by bracketing the researcher’s prior assumptions. Unlike other designs, like interpretive phenomenology, which tries to generate interpretive meanings, or grounded theory, which aims to develop theory, descriptive phenomenology fits with the study’s objective of exploring the shared essence of participants’ experiences of living with the chronic disease condition [28–30].

**Study area:** The study was carried out in Patan Hospital. Patan Hospital provides treatment and management of Type 1 diabetes through a special program and serves as one of the primary centers of the ‘Life for a Child’ program of the International Diabetes Federation [31].

**Study duration:** The study was conducted over 6 months, from 4 August 2021 to January 2022.

### Study population

**Inclusion criteria:** Patients aged 15 years and above, diagnosed with Type 1 diabetes for at least one year, according to the outpatient records of Patan Hospital, were included in the study. For the age group 15–17 years, those providing assent and whose parents provided consent were included. For the age group 18 years and above, patients who provided consent were included.

**Exclusion criteria:** Patients diagnosed with psychiatric or other neurological diseases were excluded from the study.

**Sampling procedure and sample size:** Type 1 diabetic patients who met the inclusion criteria were purposively selected for the study. The participant list was taken from the Medicine and Pediatric Outpatient Departments. The researchers included people from different backgrounds to draw rich information about the experience of living with the disease. Researchers decided to stop the process after 22 in-depth interviews (IDIs) due to information saturation, which was defined as the point where no new themes, categories, or significant variations in experiences were emerging. By the 20^th^ interview, most responses became repetitive, and by the 22^nd^ interview, no additional insights were identified, confirming saturation. Confirmation of the saturation was done by the discussion between the team members and validation with the qualitative research expert.

**Data collection tools and techniques:** An IDI guide was used for the interviews. It was first developed in English and then translated into Nepali by the researcher (APA). The translated guide was finalized after thorough discussion with a qualitative research expert (SB). The tool was pretested on two patients with Type 1 diabetes from another hospital in Nepal. In-depth interviews were conducted through telephone calls with patients at home. This mode was chosen because the study took place during the COVID-19 pandemic. Each interview lasted 20–40 minutes. Iterative questioning, probing, and debriefing were applied throughout. The researcher (APA) also advised interviewees to ensure privacy so that open conversation could occur around sensitive issues like stigma. Notes were taken on additional remarks, voice changes, or hesitation during interviews. Extraneous factors such as background noise and interruptions by family members were also documented.

**Data processing and analysis:** Audio recordings from the researcher’s phone were transferred to a computer and secured with password protection. Interviews were transcribed, translated, and imported to the R software, where the R package for Qualitative Data Analysis (RQDA) was used for data management. Colaizzi’s descriptive phenomenological method was used for the data analysis [32]. This method consisted of seven steps. First, each transcript was read several times to gain familiarity with the overall content. After understanding the whole content, significant statements were extracted from the transcript in the second step. Third step, the meanings of these statements were formulated. Next, significant statements were clustered into themes. In the fifth stage, researchers merged all the emerging concepts into a comprehensive description of the phenomena. The sixth stage involved describing the fundamental structure of the phenomena by compressing results to avoid duplication. Finally, in the seventh step, researchers returned to participants for member checking (P03, P15, P17). They confirmed through phone conversation that the transcripts and codes accurately reflected their experiences. This way, emphasis was given to describing the essential structure of the lived experiences rather than producing interpretative meanings or themes.

**Validity and reliability:** Lincoln and Guba’s constructs of rigor were used to ensure the trustworthiness of the study [33].

**Credibility:** Tools for the study were developed through an extensive literature review, and the content validity of the tool was ensured through discussion with a qualitative research expert. A detailed discussion was held with the participants, encouraging them to share their experiences. Iterative questioning was done, and frequent debriefing sessions were conducted among the team members to ensure everything was done according to the prior plan of action. The collected and analyzed data were read out to three of the participants, who confirmed that the narrative was accurate and a true reflection of their experiences.

**Dependability:** Intercoder reliability (ICR) was done independently to evaluate the reliability of the generated codes [34]. The codes were subsequently discussed and reviewed to achieve agreement between the coders. A total of 86.96% of the codes were agreed upon between the two coders.

**Transferability:** The findings of the study were clearly stated, and sufficient background information was provided to establish the context of the study. A detailed description of the research process was provided to facilitate comparisons and future use, hence ensuring the transferability of the study.

**Confirmability:** Non-verbal cues during the interviews were noted. Researchers also included their experience of the study while writing the reflexivity. Personal beliefs, assumptions, and experiences were documented, while bracketing was employed to minimize bias. Researchers only acted as facilitators during data collection. Previous knowledge, presumptions, and beliefs of the researchers were excluded from the analysis; it was strictly based on the statements of participants.

**Ethical considerations:** Ethical clearance was obtained from the Institutional Review Committee- Patan Academy of Health Sciences (Ref: PHP2108031563). Verbal informed consent and assent were taken from the study participants before conducting the interviews. For the participants of the age group 15–17 years, the purpose of the study was explained to both the participants and their parents. Assent was obtained from the participants, and consent was obtained from their parents. For participants aged 18 years and above, informed consent was obtained directly from the participants. The entire process from explaining the purpose of the study to obtaining consent and assent was audio-recorded prior to each in-depth interview, with each participant assigned a unique ID. All the participants were informed that participation was voluntary, and they had the right to withdraw from the interview at any time if they felt uncomfortable. They were also informed that there would be no direct benefit or harm from their participation in the study. The method of telephone interview, along with obtaining verbal consent and assent, was approved by the Institutional Review Committee of Patan Academy of Health Sciences. The identities of the participants were kept confidential by not recording their names anywhere in the study. They were coded with the alphabet ‘P’ and numbers (one to twenty-two). Data storage was done on the laptop of the researcher and with password protection.

## Results

### Basic information about the participants

Twenty-two participants, of whom 13 were male, were interviewed in the study with a mean age of 23.32 years (range: 15–44 years). Twelve of the participants were from the Kathmandu Valley, two were from the adjacent peri-urban areas of Thankot and Kritipur, and the remaining eight were from further outside this city. Fourteen participants had completed the School Leaving Certificate (SLC) and above, six were in some secondary, and two were at the primary level. Twelve participants were employed, among whom seven were involved in grocery shops, garment stores, and agriculture. Others were electricians, dental assistants, painters, bus staff, and a yoga teacher. Everyone had at least one sibling, with the majority having two. Two of the participants had a family history of Type 1 diabetes. Seventeen among the total participants were doing the HbA1c test regularly, among whom seven had an HbA1c level below seven, and the remaining had a higher range. Key parameters are summarized in the table below (Table 1).

**Table 1:** General characteristics of the participants.

S.N	Codes	Place of Residence	Age (completed years)	Sex	Educational Level	Years of diagnosis	Recent HbA1c Level in percentage
1	P01	Okhaldhunga	15	Male	Some secondary	3	Doesn’t monitor
2	P02	Pokhara	21	Female	SLC and above	11	5.8%
3	P03	Thankot	20	Male	Some secondary	9	11.7%
4	P04	Kabhre	28	Male	SLC and above	15	Doesn’t monitor
5	P05	Lamjung	21	Female	SLC and above	9	9%
6	P06	Ramechhap	16	Male	Some secondary	7	Doesn’t monitor
7	P07	Lalitpur	24	Male	SLC and above	14	6.1%
8	P08	Ramechhap	28	Male	SLC and above	17	9%
9	P09	Kathmandu	15	Female	Some secondary	7	8%
10	P10	Kathmandu	26	Male	SLC and above	8	Doesn’t monitor
11	P11	Bhaktapur	15	Female	Some secondary	12	7.8%
12	P12	Udayapur	22	Female	Primary	13	Doesn’t monitor
13	P13	Lalitpur	16	Male	SLC and above	15	8.5%
14	P14	Kathmandu	26	Female	SLC and above	18	6%
15	P15	Kathmandu	22	Male	Some secondary	8	Don’t remember
16	P16	Lalitpur	20	Female	SLC and above	11	6.3%
17	P17	Kathmandu	19	Male	SLC and above	18	8.7%
18	P18	Ramechhap	34	Male	Primary	16	8.3%
19	P19	Kathmandu	28	Female	SLC and above	19	5.8%
20	P20	Lalitpur	44	Male	SLC and above	12	6.5%
21	P21	Kritipur	28	Female	SLC and above	11	7.8%
22	P22	Kathmandu	25	Male	SLC and above	9	6.4%

*Response categories of educational level adopted from Nepal Demographic Health Survey, 2016.

### Common patterns and elements of the lived experience of Type 1 diabetes patients

Initial codes extracted were revised and refined multiple times. Forty-six codes were identified at the end of the revision. The identified key elements having common overriding patterns were identified and listed together, resulting in the themes of the study (Table 2).

**Table 2:** Identified themes and their codes.

S. No.	Theme	Subtheme	Codes (or Elements)
1.	Initial hospitalization		Symptoms Diagnosis Serious health condition Young age of onset
2.	Solidarity in illness and support systems		Initial feelings Type 1 diabetes group Family support
3.	Learning process and acceptance		Learning from health personnel Gradual learning Sources of information Development of skills Positive self-esteem
4.	Changes and adjustments		Food habits Travelling Patterns Career choices Balanced lifestyle
5.	Diabetes management challenges		Insulin challenges Struggles with hypoglycemia School college difficulties Challenges related to managing food when with friends Lack of motivation for exercises
6.	Concerns and worries		Diabetic complications in the future Concerns related to future career impacts Worries related to disease in children Parental concerns
7.	Socioeconomic and structural challenges	Socioeconomic challenges Structural challenges	Lack of knowledge in the school college setting Difference between Type 1 and Type 2 diabetes Lack of awareness Free services Monthly expenses and follow-up Financial strain placed on the family Foreign studies and employment Travelling Sports and fun activities Quality of healthcare available at their place Cannot visit in shorter period Unavailability of necessary medicine and other items
8	Healthcare experiences		Experience related to healthcare workers Satisfaction related to the treatment procedures
9.	Type 1 diabetes-related stigma		Disclosing diabetic status Taking insulin in front of others Unnecessary societal attention
10	Experiences of living with Type 1 diabetes during the COVID-19 pandemic		Missed follow-ups Continuation of everyday life Vaccine hesitancy Availability of medicine and other necessary items

### Initial hospitalization

Experiences included the process and pattern of the participant’s initial hospitalization, along with the description of their journey toward being diagnosed with Type 1 diabetes**.** Most patients reported experiencing common symptoms of diabetes. They recounted being diagnosed in a different hospital before being referred to the Patan Hospital for further care. Additionally, five participants described visiting a shaman prior to arriving at the hospital.

*“The condition was very bad, we had no idea what was wrong, but instead of going to the hospital, we went for ‘fukna lai’ [a traditional way of healing]. I was unconscious by then. The shaman there said it seemed to be symptoms of sugar, and that she should be taken to the hospital.” (P16)*

A young age at the onset of the disease was found in most of the patients, and they shared being in a serious health condition at the time of initial hospitalization. Some also expressed their wish to have been diagnosed rather later in life, as an early diagnosis required many compromises.

*“In the ……, the saline water was not getting into my body from anywhere, and finally, they had to infuse it through my forehead. I was... in the hospital for one month in total and in the ICU for 7 days.” (P13)*

### Solidarity in illness and support systems

Participants shared their feelings about the initial stage of diagnosis, their ups and downs related to the dilemma of understanding it, and the pattern in which they first reacted and later consoled themselves. Most participants said they were scared, as the sudden onset of the disease was a terrible experience, and its complexity was difficult to understand. Being in a group served as the primary coping mechanism for many. They explained that upon arriving at the Patan Hospital and seeing other individuals in similar situations, they felt relieved as they were not alone, and if others could manage, so could they.

*“As I started to visit ……Hospital, I met many people with the same diagnosis, and we became friends. I even came across a 2-month-old baby with diabetes. And after that, I felt it was not a big disease to stress about and take tension.” (P21)*

The family was identified as a major support system by the participants. Experiences of having adequate support from their families were shared.

*“They made sure I had everything I needed from the beginning. They created an atmosphere in which I never felt like I was unwell. They were always there……. My brother, for example, dropped his examinations because I needed care when I became ill.” (P14)*

### Learning process and acceptance

Most participants shared that the concept of diabetes self-care management was introduced to them or their family members by healthcare workers during their hospital stay. For many, learning was a gradual process over the years through personal practice. All agreed that the hospital was the primary source of their information. In addition, many turned to the internet. Some also mentioned newspapers, books, radio, and teachers as additional sources.

*“Firstly, they did it and showed the technique. After coming back home for a complete 1 year, my mother used to give me the injection, and then, slowly, I started to take it myself. And when I started, she used to ask me to take the injection in front of her for some time.” (P06)*

Participants were doing diabetic self-care management on their own, and for most, the condition was normal for now. They showed good acceptance of the condition and maintained positive self-esteem.

*“I remember thinking as a child that I wished there was a cure for this. I wish I were as normal as my buddies. But now I feel, I do have an illness, that nothing can be done…. I don’t feel anything.” (P17)*

### Changes and adjustments

Various experiences regarding how diabetes changed their lives and adjustments made to continue living with the disease were shared. All mentioned changes in their food habits. Most explained that rather than following a strict diet, they ate in moderation. They also described adjustments in their travel patterns after diabetes, and for some, carrying insulin while travelling was a major concern.

*“Now in …. travel patterns, it is difficult in terms of insulin storage. …. we come from Pokhara to Kathmandu itself, it is about a 6-hour ride, and we must take insulin with us, that is a problem.” (P02)*

Experiences related to leaving their chosen career to better manage their diabetes, believing those careers were not good for their glycemic control, were shared by the participants.

*“After SLC, I wanted to study to become a nurse. My sugar level used to be very high during those times. We all felt pressure that the studies might result in a further increase in sugar levels, which is why my family forced me to drop the idea of pursuing nursing studies.” (P14)*

Following an exact routine in daily life was not possible for most of them. They shared their experiences of adopting certain routines to balance diabetes management with everyday life.

### Diabetes management challenges

Despite their efforts, most participants were unable to avoid hypoglycemia. Those who went to school or college shared that they found it challenging to comply with diabetes management and class schedules. For some, it was difficult to find a place to take insulin. Challenges regarding sticking to food dos and don’ts when with friends were also shared. One participant recounted leaving school after the diagnosis.

*“The one thing I can’t do according to the advice is to maintain my blood sugar levels. The doctor advises eating and checking before going to bed. I always check my blood sugar level before going to bed ……. but it still goes into hypo. My family members become angry with me; ………. I feel helpless because no matter what I try, nothing seems to work.” (P14)*

*“The main problem is managing food. If there are parties such as birthday parties, I cannot go because of the food problems, and even when we go out, I cannot eat much….” (P09, 15yrs)*

Some of them lacked motivation for exercise. Pain and calculating insulin doses were additional difficulties for a few of them.

### Concern and worries

A major concern expressed was the potential development of diabetic complications in the future, with two already reporting memory loss at the time of the interview.

*“They say that as time passes and the duration of a diabetic patient’s diagnosis lengthens, the patient’s memory deteriorates. I’m starting to feel like I’m losing my memory……………. I am getting very forgetful; I just easily forget my lessons, no matter how nicely I study.” (P21)*

They also voiced concerns regarding their career prospects and future progress. Financial difficulties were noted, particularly the challenge of meeting ongoing medical expenses, and some participants feared they would be unable to cope if their children were also diagnosed with the disease.

*“Sometimes I feel that ……. what if my children also have this, what will we do then. It takes about 2-3 thousand for my treatment per month; if my children also get it, it will cost us more. How will we manage then………..” (P18)*

Constant parental concerns related to their well-being were also experienced by them, and in two cases, participants shared their parental concerns related specifically to their marriage prospects.

*“Despite me being a teenager and an adult, their problems persist. Currently, they are having problems regarding my marriage. (Laugh) Getting their daughters married is considered an important responsibility for parents. My family, especially my mother, is worried about my marriage prospects……. They cannot marry me off without telling the groom’s party about my disease, so concerned ……?” (P19)*

### Socioeconomic and structural challenges

This theme explores the multifaceted challenges faced by individuals with Type 1 diabetes in Nepal. Participants described difficulties related to financial strain, awareness gaps, restricted opportunities, and disparities in healthcare access.

- Socioeconomic challenges

Participants reported considerable financial strain due to the ongoing costs of insulin, follow-ups, and hospital visits. Although the provision of free insulin and related materials at Patan Hospital until the age of 25 was beneficial, financial pressure increased thereafter. In some instances, insufficient funds forced respondents to forgo essential diagnostic tests. Awareness gaps were also prominent. Teachers often lacked the knowledge to manage diabetic emergencies, leading to the exclusion of students from school activities. Furthermore, misconceptions between Type 1 and Type 2 diabetes underscore the need for awareness programs, particularly in rural areas.

*“Besides medicine, sometimes there were incidences of my blood sugar being low and high requiring hospitalization, ……. created an added financial burden. Constant expenses were there for medicine and blood tests, and such incidents further added……., resulting in my family members thinking about how they will manage or them feeling like they might land in a serious financial crisis.” (P19)*

*“………, I want to say to you that we are all the new generation. We are not ……do much help to others with Type 1 diabetes, but we expect awareness to spread in the village areas. Doctors should remain in the village and help the children with Type 1 diabetes.” (P22)*

- Structural challenges

Some of the participants were unable to pursue opportunities abroad for study, work, or training because of parental restrictions, personal reluctance, and, in some cases, government regulations. The participants residing outside the Kathmandu Valley experienced additional challenges, including limited hospital visits and concerns about the quality of healthcare services available in their respective regions.

*“My family did not use to send me anywhere; constantly they would say you are sick…. that continued, resulting in overprotection and over-care by the family. When it came to job opportunities, there too my family objected ……, you will go for one day then get sick for 10 days, so I missed many opportunities……….” (P21)*

*“There are hospitals in my place, but they do not provide you with treatment as expected. They admit you here even if you have a fever and then refer you somewhere else 4-5 days later. They diagnose you with one illness and then refer you to ……. where you get diagnosed with another. Hence, I have trust issues with the place here.” (P08)*

### Healthcare experiences

Most participants were happy with how the healthcare workers treated them, describing them as helpful and understanding. A few complained about inadequate counseling. Satisfaction related to the ongoing treatment procedures was expressed, though some wished they could switch to tablets.

*“They behave very nicely. They soothe and encourage me by saying this is not a big, dangerous illness.” (P01)*

*“Sometimes I wish that we could just use medicine instead of injection.” (P11)*

### Type 1 diabetes-related stigma

Some participants reported difficulties in disclosing their diabetic status to others, such as in-laws, maternal uncles, or colleagues in the workplace. They often sought a quiet place to administer insulin. Participants also described distressing experiences of receiving unnecessary attention from relatives and neighbors. A few further explained that they dealt with this situation by ignoring it.

*“I don’t feel uncomfortable in front of my family members, but when I go to my in-laws’ place or my maternal uncle’s place or any other relative’s place…., I feel uncomfortable then.” (P18)*

*“I ignore these things. My life is like this, so I must live like this and ignore those things; it is useless to pay attention to those things.” (P15)*

### Experiences of living with Type 1 diabetes during the COVID-19 pandemic

The COVID-19 pandemic disrupted diabetes care, as lockdowns and social isolation negatively affected patient follow-ups. Most of them reported missed appointments, and for some, it had been up to 2 years since their last visit. Many who had returned to their villages during the lockdown, as well as those from outside the Kathmandu Valley, shared difficulties in obtaining insulin and other essential supplies during the height of the pandemic. Also, a few of them were hesitant to take the COVID-19 vaccine.

*“During the first wave, it was difficult for us. No public vehicles were running, and we do not own any. …….. In that condition, if I had to reserve a vehicle, it would cost about Rs 5000 for going to Kathmandu only and Rs 10000 for two ways…. I knew an ambulance driver, and he helped to get the medicine after I explained the situation.” (P08)*

*“It was difficult to manage everything. Having insulin was not enough; we also needed the syringe and the other kinds of stuff. ……..” (P05)*

## Discussion

This study portrays delayed hospitalization, solidarity in illness, and various support systems, a gradual learning process, changes and adjustments brought by the diagnosis, various challenges faced, concerns and worries, healthcare experiences, Type 1 diabetes-related stigma, and experiences during the COVID-19 pandemic faced by the patients living with Type 1 diabetes. The study aimed to explore the essence of the experiences lived by Type 1 diabetes individuals in the Nepalese context. A young age of onset and serious health conditions were reported, consistent with prior studies from Patan Hospital and the Eastern Region of Nepal, where diabetic ketoacidosis was a common initial presentation [25,26]. The sudden onset and the lifelong nature of the disease were emotionally distressing to the participants, consistent with previous studies’ findings [11, 35,36] Familial support was critical for adjustment and acceptance, corroborating evidence from Sweden [35].

Confusion between Type 1 and Type 2 diabetes highlighted widespread knowledge gaps, reinforcing the need for public awareness initiatives. These issues are consistent with research from Sweden and Nepal, where the need for community awareness was emphasized [24,26,35]. Peer group participation further facilitated coping, aligning with findings from Ireland that demonstrated the role of social support in mitigating stress [37]. Participants reported gradual self-learning in diabetes management, including dietary regulation, portion control, insulin administration, and glycemic control. This aligns with the study done on the development of learning patterns, which identified a gradual learning pattern shaped by support, knowledge, barriers, and coping skills [38]. Healthcare workers were the primary source of information, supplemented by the internet, newspapers, books, radio, and teachers [39,40]. The gradual development of self-care skills in managing hypoglycemia and hyperglycemia corroborates King et al.’s findings, which emphasize the steady internalization of knowledge and skills among individuals with Type 1 diabetes [36]. Participants emphasized being self-sufficient in insulin dosing, diet, and blood glucose testing, echoing themes reported in a study from Denmark [14]. Furthermore, most participants exhibited positive self-esteem, accepting their condition well, and viewing it as a normal part of life. This is consistent with a Canadian study, which found that adults with Type 1 diabetes faced their condition with resilience, adaptability, and a sense of personal accomplishment [3,41,42].

Lifestyle changes focused on dietary modification with moderation as the guiding principle rather than strict dieting. They also reported modifications in travel patterns and career choices to better manage their diabetic status. These findings are consistent with previous studies, which described the impact of diabetes on daily life, requiring adjustments to fit care into routines [35,36,43]. Similar themes were echoed in the lived experiences shared by family members of individuals living with diabetes [9]. Participants preferred guidance on moderate eating and carbohydrate control instead of strict restrictions, describing it as easier to follow. This approach is similar to an Australian study’s conclusion emphasizing individualized diabetes care [44].

Fear of hypoglycemia, especially at night or when alone, was common among participants, consistent with previous studies on Type 1 diabetes [14,36,45,46]. Concerns about being a lifelong burden and the need for constant vigilance were also noted in the study; findings are similar to other studies done in the family members of Type 1 diabetes [9,47]. Students faced difficulties managing diabetes with school or college schedules, particularly morning classes, due to insulin and meal timing. Unlike a Swedish study where uncooperative teachers were the main issue, contextual factors such as school meals availability may explain this contrast [35]. Participants reported low motivation to exercise, largely due to a lack of time and viewing daily work as sufficient activity, similar to UK findings [48]. Injection pain and difficulties adjusting insulin during illness or hyperglycemia were frequent challenges, consistent with Indian research [16]. Social situations complicated dietary adherence, consistent with existing literature [49–53].

Concerns about diabetes control, genetic transmission, and effects on children’s health and life were consistent with earlier studies [14,36,54,55]. Concerns also involve limitations in career opportunities and future aspirations, showing the broader impact of diabetes on various life domains [55–58]. Parental concerns regarding children’s health, safety, and marriage aligned with studies on persistent family anxieties [35,59]. Financial burdens, including the cost of medications, follow-up visits, and supplies, were in line with Nepalese, Gujarati, and Southern Indian studies [25,26,60]. School barriers included poor teachers’ understanding, exclusion from trips, and inadequate emergency management, consistent with the Swedish study [35]. Participants expressed a preference for oral medication over insulin and hope for an ultimate cure, similar to findings in related research [24,61–63]. Restrictions on foreign travel, school activities, sports, and career choices, often due to parental or personal reluctance, reflected a desire for autonomy in decision-making about life activities as noted in a Danish study [14]. Those outside the Kathmandu Valley faced additional challenges consistent with evidence from other studies conducted in Nepal, including Sindhuli and Karnali [64,65].

Stigma was another concern. They often felt discomfort disclosing their condition or administering insulin in public, consistent with Australian findings of unwanted attention and emotional distress [66]. Similar findings were also reported in other studies [43,22]. The COVID-19 pandemic further contributed to challenges, with many missing follow-up visits and struggling to access insulin and supplies. These difficulties align with reports from Rwanda, where 74% reported disruptions to diabetic care, though medicine access was less affected [10]. Vaccine hesitancy, largely driven by fear and misinformation, was also seen, consistent with findings from Saudi Arabia [67].

One unique finding was the participants’ experience of financial relief through hospital-provided free medicines and supplies up to the age of 25 years, with participants expressing a desire for this support to be extended to older age groups. Participants outside the valley suffered healthcare inequalities. They lacked adequate access to quality healthcare services outside Kathmandu Valley, necessitating visits to the capital for each follow-up and other services.

The study has some strengths and limitations. To our knowledge, this is the first systematic and in-depth phenomenological examination of the lived experience of living with Type 1 diabetes in Nepal. This study’s strength includes its relatively innovative topic and the data gathered comprehensively through in-depth interviews. Study design and measures taken to maintain the rigor of the study, like verification of the transcript and translation scripts, member checking, inter-coder agreement, and process documentation, further add to the strength of the study.

Phone interviews turned out to be a limitation, limiting the researcher’s ability to observe participants’ facial expressions and body language. Disruption sometimes occurred due to poor network connectivity, requiring repetition of questions and answers, potentially affecting the depth of some interactions. Furthermore, being a phenomenological study, the findings were primarily based on participants’ ability to share their experiences, which may not adequately represent the wider population of individuals with similar conditions.

This study focused on Type 1 diabetes patients who had been diagnosed at least a year before the interview. The participants highlighted difficulty coping in the initial stage with gradual acceptance. Future research should focus on newly diagnosed patients, adolescents, family perspectives, and the long-term psychosocial impacts of Type 1 diabetes in Nepal. This may lead to packages of care catering to the entire biopsychosocial spectrum of the disease. Type 1 diabetes-related stigma requires more attention in the future. The impact of emergency conditions like COVID-19 on the quality of life of Type 1 diabetes patients also requires further research. Additionally, an assessment of knowledge about the differences between Type 1 and Type 2 diabetes and their respective management strategies should also be done.

## Conclusion

Findings of the study emphasize the need for accessibility of healthcare services, particularly in rural areas. Emergency conditions like the COVID-19 pandemic further intensified these challenges, leading to missed follow-ups and difficulties in accessing essential medicines and supplies. This study emphasized the urgent need for comprehensive community awareness programs, especially in rural regions of Nepal, to address the knowledge gap about Type 1 diabetes. Type-1 diabetes-related stigma, a lesser-discussed issue in Nepal, should be addressed through mass media, campaigns, and hospital-based health education programs. The study recommends extending the age limit for free diabetes services and supplies, as well as expanding programs like “Life for a Child” to at least one center in each province. The School Health Nurse program implemented by the Government of Nepal should be strengthened to support students with diabetes in educational settings. Regular classes or reinforcement programs on Type 1 diabetes self-care should be conducted in the hospitals to ensure compliance and motivation. Periodic counseling sessions for parents are also important to prevent overprotection. The participants highlighted learning from and being inspired by fellow Type 1 diabetics, indicating the value of establishing patient-support groups. Finally, the study calls for improved emergency preparedness from the government and all the responsible stakeholders to ensure uninterrupted healthcare access for chronically ill patients during a crisis such as COVID-19.

## Supporting information

S1 Table Themes with their verbatim. This table contains additional verbatim quotations for each identified theme, providing further information to support the qualitative findings.
(DOCX)

S1 Appendix Questionnaire. (DOCX)

S2 Appendix Information sheet, informed assent, and consent form. (DOCX)

S3 Appendix Reflexivity. (DOCX)

S4 Appendix Excerpts of the interviews. (DOCX)

S1 File Sample interview transcript 1. (DOCX)

S2 File Sample interview transcript 2. (DOCX)

S1 Checklist COREQ checklist. (DOCX)
