# Supplementary material for: Lived experiences of Type 1 diabetes patients visiting a tertiary care hospital of Nepal: A descriptive phenomenological study
Source: PLOS Glob Public Health. 2026 Jan 13;6(1):e0005810. doi: 10.1371/journal.pgph.0005810 (PMC12798998; doi:10.1371/journal.pgph.0005810)
Supplement: S1 Table — This table contains additional verbatim quotations for each identified theme, providing further information to support the qualitative findings. (DOCX) [file pgph.0005810.s001.docx]

**Table: Themes with their verbatim**

| Theme: Initial hospitalization*“I started feeling thirsty frequently and also had to pee a lot and when I went for a checkup my sugar level was 652 initially.” (P07)* *“I was sick for a long period. I went to a nearby clinic several times for check-ups, and during one visit they told me that this was due to weakness and gave me vitamins. After that thing became increasingly difficult, so we sought second opinions from several different clinics, and finally went to …. Hospital, where they performed various blood tests and informed us that I had diabetes.” (P19)*  *“They took me to the shaman to get me checked if something had happened to me, as that is what's done in the villages. After the shaman checked me, he should have said it if he could not find the exact problem but what he said is that 'there is some wrong occurrence in the ‘selroti’ [a traditional homemade ring-shaped sweet rice bread] and some yellow substance. Whatever is found at present in Chaitra, a ripe fruit, oh there is papaya available, and make selroti and eat them after 'fukera’ [a traditional way of healing]. Then I came and told my mother that he had told me to eat 'fukera' and I ate selroti and papaya. The next day, I was not even able to sit upright because of vomiting. Then my father took me to the medical.” (P14)*  *“I am not sure how many years; I was about 3 years old when I got diagnosed.” (P11)*  *“‘Dekheko na hudo raicha lekheko chai hudo raicha’ [ In our life, we get what we are destined for every desire cannot be fulfilled].7-8 years back I used to feel like why I had to get this disease, many of my dreams were shattered. Even if I had to get it why couldn’t I have gotten it later in life when I have settled down and made my future.” (P14)* |
| --- |
| Theme: Solidarity in illness and support systems  *“About the feeling, I had heard about it, diabetes as a sugar disease, but did not know in detail. I had only heard about it a little. It had not happened to anyone whom I knew and when suddenly this out-of-the-world disease happened to me then I was stunned and very sad.” (P15)*  *“In the initial period, I did not know much about the things during my hospital stay. I used to cry because I was so scared, and the doctors consoled me saying nothing will happen. After I got discharged, I had to take insulin, and I used to cry every time I took it (Laugh).” (P19)* |
| Theme: Learning process and acceptance  *“I was told about the insulin, diet, exercises, and all during my hospital stay.” (P12)*  *“I learned with time, things are like this, this thing goes like this.” (P15)*  *“I also get information from other places too. I get from the hospital; I search on the internet for various information related to my diagnosis.” (P06)*  “*When I was diagnosed with Type 1 diabetes, I did my full research on the disease from google. I did a detailed study from Google. I found out that if I maintain it properly then nothing will happen. I mostly found the information from Google.” (P22)*  *“Regarding the information, I got it mostly from Patan Hospital. Sometimes, I do a few YouTube searches where I look at videos on diabetes. Regarding what to eat and what not to eat, what activities are good what is bad, you learn these things through the radio as well, they give announcements and information.” (P18)*  *“I take care of myself; I know what works for me and do accordingly.” (P04)*  *“I share about these things when I am talking to people normally or when I am talking to a group. I am a diabetes patient, and I am strong is the first thing I tell them.” (P14)* |
| Theme: Changes and adjustments  *“I have been strictly avoiding sugary products and potatoes. Other than them there are not many items that I avoid.” (P21)*  *“Initially, they gave us a pattern to follow for eating, we had to eat according to the schedule and that was difficult but now I manage myself and eat in moderation. Food items also I use one in the replacement of other according to my circumstances, now it has become easier.” (P02)*  *“Constant concerns related to the misbalance of the diet while traveling, lack of a place to store insulin, and issues related to where to take it. Having to carry and take insulin while traveling is a bit uncomfortable. After being diagnosed I have to take insulin regularly throughout my life, so this is also a change or added tension”. (P19)*  *“Sometimes I feel bad. I could not try for the army or police but it's like this what to do.” (P17)*  *“Despite my long working hours, I have balanced my routine. I take my food in time. It is only sometimes when there is much work pressure, I become late, but it only happens sometimes. I always try to maintain a balance.” (P22)* |
| Theme: Diabetes management challenges  *“………… And if it happens while you are sleeping you won't find out and so my family needs to be extra careful because of that I feel I will always be a burden to my family.” (P14)*  *“But sometimes when I go out and suddenly, I get into hypo at that time I feel like what kind of illness is this that I am going through because when you get into hypo you sweat a lot and if you don’t find food anything can happen and at that moment, I feel what kind of illness did I get.” (P20)*  *“We have a fixed routine for the timing of insulin. When it is time to take insulin-like at 8 o’clock, I have to go to the bathroom to take it. There is no separate place in the classroom or anywhere in the college and that makes it difficult. And it is challenging as my classes are in the morning. Time management and following the routine is a huge problem.” (P13)*  *“I don’t do any work or go around. I just stay at home.” (P05)*  *“I don’t know why, but I did not feel like going to school so I left my studies. (Laugh) I left my studies after my diagnosis.” (P12)*  *“I used to exercise before but now I don’t. Now, as my sugar level is fine, I feel I don’t have to do much (laugh) I also feel lazy, and that’s why (laughs).” (P16)*  *“I do not do exercise separately. I exercise by doing household work.” (P18)*  *“The injection …… was painful and had to be injected near the umbilicus and thighs, it was not only for one day but had to be done twice daily, once in the morning and once in the evening. It is difficult to keep injecting and making holes everywhere. It used to hurt even to touch after all the injections.” (P10)*  *“It is difficult to adjust the night doses rather than that lespro is not that difficult. Lantus is a little difficult.” (P09)* |
| Theme: Concerns and worries  *“I worry about if my eyes will be affected, my kidney will be affected.” (P03)*  *“I wonder if people will dislike me because of my disease, whether this disease will create a barrier in my career, I worry this disease will hold me back. Since my parents' financial condition is poor, I worry about what will happen if I cannot control it. I worry this disease might hinder my progress.” (P13)*  *“My family does that; they frequently inquire about my whereabouts when I am not in the house, or I am late.” (P18)* |
| Theme: Socioeconomic and structural challenges  *“In terms of financial hardship, there is an additional burden because insulin is costly. As a result, you'd have to spend a lot of money on medicine. I don't have my source of income; everything I do is from the money of business, so there's tension because I sometimes feel obligated to remain dependent (laughs). That's it; because I don't have employment, I have to rely on my husband for medicine.” (P21)*  *“I have this kind of disease, and I am getting free medicines, so I am happy. If I had to buy it all by myself then it would be difficult.” (P15)*  *“There are many expenses and added financial burdens. It is difficult in villages as we don't have much money. For one visit to the hospital, you must take about 8-10 thousand rupees for travel and hospital expenses. You must take the HbA1c test along with Urine tests and other tests. There are times when I have returned from …. hospital to my village without doing those tests as I did not have enough money.” (P18)*  *“There are financial problems sometimes. Sometimes we don’t have money, and it becomes even difficult to get check-ups.” (P03)*  *“During school when we had trips teachers used to say that I don’t have to go.” (P03)*  *“When I was in class 9 ………, during class my blood sugar became low, and I was sitting on the last bench. I tried calling the teacher, but my voice was low, my heart was shaking, I was sweating, and I was about to faint, so I took a juice out of my bag and drank it, the teacher was watching me apparently, she came over ……and yelled at me for eating during class. I told her my sugar level became low but she ……… and scolded me. I felt very hurt by this incident, and I cried.” (P14)*  *“We are told we have Type 1 diabetes and there are Type 1 diabetes, Type 2 diabetes, and Type 3 diabetes, what are those? I have not understood these things much to date.” (P08)*  *“I feel in Nepal there is a huge lack of awareness on this topic, in our villages. While collecting data, I came across an incident where a 12–13-year-old girl was kicked out of her house because she got diagnosed with the disease, and the family was concerned they would have to look after her and provide her medicine for her whole life. There is a need to spread awareness about this disease in rural areas. I feel awareness campaigns on Type 1 diabetes must be launched in rural areas.”(P14)*  *“I think about my friends, they have achieved so many things, on the other hand, I am sick so I cannot even openly go around with them if we have to go somewhere together or go to a new place it is difficult for me.” (P15)*  *“There are so many opportunities that come in our school, they take you on trips. Such as for singing they take you to many places for training and I am unable to go.” (P09)*  *“There are some incidents related to missed opportunities. Once I had got an opportunity to go abroad for a program, but my family did not allow me to go because of diabetes.” (P19)*  *“After my 12, I went in search of language classes and also for information about the scholarships in Japan, but because of my diabetes, they said it cannot happen and was turned back (laugh).” (P16)*  *“The doctor taught me all these things. He taught me these things as my house is far from Kathmandu and must manage these conditions on my own as I cannot rush to the hospital every time regarding the fluctuations in the blood glucose level until and unless it is a serious condition.” (P18)*  *“Routinely I used to do my blood sugar test at home, but since the kit is finished and it is not available here, I have not been able to do that now. I have to go to Kathmandu to find them.” (P08)* |
| Theme: Healthcare experiences  *“They don’t give you any such counseling, they just give medicine.” (P03)*  *“The treatment is good. I also take references from the net sometimes. Type 1 diabetes is a dangerous one, you must take insulin. I take it as there are benefits of taking insulin too. It is fine.” (P08)*  *“I wish I could take medicine instead of the insulin.” (P03)* |
| Theme: Type 1 diabetes-related stigma  *“Outside of college, I don’t tell anyone I have this, I still don’t tell people about my diabetic status because I don’t want them to dominate me by telling them I have this.” (P19)*  *“I fear people will dislike me and mistreat me because of this disease.” (P13)*  *“In terms of uncomfortableness when I go to new places as guests and they serve you food, I have to disappear for about 15-20 minutes when they say it is meal time, during that time I have to go find a quiet place and take the insulin, I feel a bit awkward at that moment disappearing 15-20 minutes before food all of a sudden. (Laughs).” (P18)*  *“It is difficult to tell friends openly about the disease. Sometimes when they come to know, they just do unnecessary gossips about me like he has this and that, it feels bad, really bad.” (P15)* |
| Theme: Experiences of living with Type1 diabetes during the COVID-19 pandemic  *“Before I used to go for follow-up every 3 months, but I have not gone since COVID-19.” (P04)*  *“The vaccine came, but we were not sure if I could take the vaccine or not, we cannot go like others and just say let’s take it, we need to be a little cautious.” (P02)*  *“We were told to get vaccinated, but we were also told that we should not take the vaccine. We were all in doubt and were hesitant to take the vaccine.” (P05)*  *“I have heard that even a healthy person gets sick after getting the vaccine is it right? I worry that we sick people might get sicker because of this vaccine. Many of the people here got sick after getting the vaccine. I was worried that since I am already sick if I get vaccinated, I might be paralyzed, and it might cause more problems.” (P08)* |
