# Supplementary material for: Lived experiences of Type 1 diabetes patients visiting a tertiary care hospital of Nepal: A descriptive phenomenological study
Source: PLOS Glob Public Health. 2026 Jan 13;6(1):e0005810. doi: 10.1371/journal.pgph.0005810 (PMC12798998; doi:10.1371/journal.pgph.0005810)
Supplement: S1 Appendix — (DOCX) [file pgph.0005810.s002.docx]

## Questionnaire (English)

**Introduction**

Participant’s ID………..

Date…………………………………….

| SN. | Category | Coding Answer | Skip To |
| --- | --- | --- | --- |
| Part I: General characteristics of the participants: | | | |
| 1. | Place of residence |  |  |
| 2. | Age (Completed) |  |  |
| 3. | Sex | 1. Male  2. Female |  |
| 4. | What is your educational level?* | 1. No Education 2. Primary 3. Some Secondary 4. SLC and Above |  |
| 5. | What is your occupation?(if applicable)* | 1. 1.Agricultural 2. 2.Clerical 3. 3.Sales and services 4. 4.Professional/ technical/ managerial 5. 5.Skilled manual 6. 6.Unskilled manual 7.Others |  |
| 6. | Do you have any siblings? | 1. Yes 2. No | If no go to q.no.8 |
| 6.1. | How many siblings do you have? |  |  |
| 7. | What is your birth order? |  |  |
| Part II: General information on Type 1 diabetes status of the participants: | | | |
| 8. | How long has it been since the diagnosis? |  |  |
| 9. | Do you have a family history of Type 1 diabetes? |  |  |
| 10. | Do you get your HbA1c levels checked regularly? | 1.Yes  2.No |  |
| 10.1 | If yes what was your most recent HbA1c level? |  |  |

(*Note-Response Categories of educational level and occupation adopted from NDHS, 2016)

**In-depth Interview Guide**

| SN | In-depth Interview Guide |
| --- | --- |
|  | How did you come to know that you have Type 1 diabetes? How was your medical condition at the time of diagnosis? Please share your full story. |
|  | Can you describe in detail your feelings in the initial period? What did you do at that time? Please share your experience with us. |
|  | After being diagnosed to present day, describe your experience related to diabetes self care management? How did you learn it? Please share your experience with us. |
|  | How has the diagnosis changed your life? How do you cope with it? Do you share your experience with others to help them to cope with the condition? Please share your experience with us. |
|  | How has the diagnosis changed the life of your family? Please share your experience with us. |
|  | What aspects of your diagnosis worry you the most? How do you see your future? Please share your experience with us. |
|  | Regarding the components of diabetic self-care management, which components are giving you hard time? What are the challenges related to compliance with the treatment regimen? Please share your experience with us. |
|  | Describe in detail your experiences with the health care facilities and the health care personals? Are you satisfied with your current treatment? Please share your experience with us. |
|  | What are the things according to you, Type 1 diabetes has prevented you from achieving or doing**?** Please share your experience with us. |
|  | Have you experienced any type of discrimination or stigma related to your disease condition? How have you been dealing with it? Please share your experience with us. |
|  | Please share your experience of living with Type 1 diabetes in this pandemic (COVID-19). |
|  | I asked you lot of questions regarding your experience of living with Type 1 diabetes and took lot of your time and in some moments I may have made you emotional. Regarding the conversation, is there anything missing? Is there anything you want to share further with us? |

**Thank you for your time**
