# Supplementary material for: Lived experiences of Type 1 diabetes patients visiting a tertiary care hospital of Nepal: A descriptive phenomenological study
Source: PLOS Glob Public Health. 2026 Jan 13;6(1):e0005810. doi: 10.1371/journal.pgph.0005810 (PMC12798998; doi:10.1371/journal.pgph.0005810)
Supplement: S2 Appendix — (DOCX) [file pgph.0005810.s003.docx]

## Information Sheet

**Research Title: Lived Experiences of Type 1 Diabetes Patients Visiting a Tertiary Care Hospital of Nepal: A Descriptive Phenomenological Study**

Namaste,

My name is……………………… I am a student from Patan Academy of Health Sciences, Lalitpur. I am undergoing Masters’ Degree in Public health and doing this study for the partial fulfillment of the requirement of the curriculum. I humbly requested you to take part in the study aiming to explore the lived experiences of living with Type 1 diabetes among the patients visiting Outpatient department of Patan Hospital. l would like to have a conversation with you regarding your experience of living with Type 1 diabetes. Please do listen to the information sheet and in case of any doubt please ask questions before agreeing to take part in the study.

The purpose of the study is to explore the lived experiences of living with Type 1 diabetes and to find the common essence and elements of living with the disease. The period of the conversation will be around 35-40 minutes and the informations shared will not be necessarily right or wrong. The interview will include questions or situations related to your lived experiences.

The information provided will be kept confidential and only be used for the study purpose. Findings of the study may be published in scientific publications but your identity will be kept confidential throughout the process. There is neither any direct benefit nor harm in participating in the study. But your experience may be beneficial to others with a similar condition to learn to live with Type 1 diabetes. During the interview you can let me know if you require any breaks, interview will be resumed after you being comfortable. Participation in the study is completely voluntary, it depends solely on your decision. If you are not comfortable answering any questions, you can skip them and can quit the interview at any time if you wish. Your refusal will not affect the future relationship with anybody related to the study. I herby hope for your precious time and participation in this study.

Thank You

## Informed Consent (For Participants of age group 18 years and more)

I have explained you about the purpose and nature of the study. Do you still have any further doubts and queries related to the study? If yes please let me know. As the participation is completely voluntary after knowing all these details are you interested in taking part in the study?

Yes……………….

No………………….

Date

## Informed Consent Form (Parental consent form for the participants of age group 15-17 years)

My name is ……………………..I am a student from Patan Academy of Health Sciences, Lalitpur. I am undergoing Masters’ Degree in Public health and doing this study for the partial fulfillment of the requirement of the curriculum. I am conducting study on, Lived experiences of Type 1 diabetes patients visiting a tertiary care hospital of Nepal. l would like to have a conversation with your child regarding his/her experience of living with Type 1 diabetes. The period of the conversation will be around 35-40 minutes. There is neither any direct benefit nor harm in participating in the study but your child’s experience will be beneficial to others with a similar condition to learn to live with Type 1 diabetes. I assure you that information provided for this study will be kept confidential and when publishing the research findings identity of your child will remain anonymous. Participation is voluntary and your child can quit at anytime if he/she wishes to. Do you have any queries related to the study? If yes you can ask me now.

Thank You

**Informed parental Consent**

I have explained you about the purpose and nature of the study. Do you provide consent for the participation of your child in the study?

Yes….

No……

Date……………

## Assent Form (For the participant of 15-17 years of age)

My name is ……………… and I am a student of Public health at Patan Academy of Health Sciences. I am conducting study on, Lived experiences of Type 1 diabetes patients visiting a tertiary care hospital of Nepal. I am asking you to take part in the study as I am trying to learn more about your experiences related to living with Type 1 diabetes. This will take around 35-40 minutes.

If you agree to be in this study, you will be asked to take part in an interview related to your experiences of living with Type 1 diabetes. Your participation in the study will not result in any instant benefit or cause you any harm. Your experience may be beneficial to others with a similar condition to learn to live with Type 1 diabetes. The continuous interview may be a problem so if you need any break you can tell me I will continue the interview after you are comfortable.

You can stop participating at any time if you feel uncomfortable. No one will be angry with you. Your information will be kept confidential. No one will be able to know how you responded to the questions and your information will be anonymous. I will also ask permission from your parents before you take part in the study. Even if your parents say “yes” you can still decide not to participate. Do you have any questions? If you are ready then we can start the interview

Yes ………………..

No……………………

Date…………………
