# Supplementary material for: Lived experiences of Type 1 diabetes patients visiting a tertiary care hospital of Nepal: A descriptive phenomenological study
Source: PLOS Glob Public Health. 2026 Jan 13;6(1):e0005810. doi: 10.1371/journal.pgph.0005810 (PMC12798998; doi:10.1371/journal.pgph.0005810)
Supplement: S3 Appendix — (DOCX) [file pgph.0005810.s004.docx]

#### **Researchers’ reflexivity**

As a researcher with a nursing background, my experience in history-taking and prior exposure to diabetic patients helped me conduct interviews. Training in Diabetic education also helped in the process. However, being new to qualitative research, I initially struggled with starting and maintaining the flow of interviews. With practice, these challenges eased after 4–5 interviews.

Instead of following a strict question sequence, data were collected by adapting to the participant’s storytelling and probing only when necessary. Purposive sampling was done for the selection of the participants but in some cases, purposive sampling turned into snowball sampling. Conducting telephone interviews posed challenges— some of the phone numbers in the list provided were either incorrect or out of service when the researcher tried to contact them. Some of the contacts of the participants were obtained through other participants. Many respondents were either students or professionals so scheduling and rescheduling of the interviews were to be done. Since the participants consisted of the age group from 15 years and above, in the 15-17 age group, family members were also present during the time of some interviews as the researcher could hear their voices from backside providing the information. Some participants provided more relevant information when supported by the family members. Some of members further facilitated participants in understanding the questions. Hesitation was found among some of them regarding sharing information, but they opened after being reassured of confidentiality. Even though the parents of some of them provided detailed information before the beginning of the interviews about the participants in informal talks, some of the participants did not provide information about those details during the interviews. Some participants even provided additional insights after the interviews ended.

As the participants were from various age groups their experiences were also different, and lost opportunities were also different. Regarding the experiences shared by the patients related to the changes done by them in their career choices after the diagnosis, it was disheartening for the researcher too to listen to their life’s realities of leaving their dreams to accompany their diabetic status. Hearing stories of young individuals adjusting their career dreams due to Type 1 diabetes was emotionally challenging. As the researcher was from a medical background many respondents asked their queries about the disease which the researcher cleared up during and after the interview and in some of the queries researcher also asked the respondents to further consult their respective consultants. Many of the phone numbers provided were of parents and after the researcher explained the purpose of the study, they further provided the contact number of the respondents.

In the study, the researcher only acted as the interviewer and tried to stay neutral throughout the process. Facilitations of the interviews were done to ensure that the participants shared all their feelings and experiences related to living with Type 1 diabetes without any hesitation. Researchers listened to the experiences shared by the participants with minimal disruptions and probing. The perception and feelings of the researcher through interactions with all the participants during the study were noted and explained above as it helped in improving the quality of research findings.
