# Supplementary material for: Lived experiences of Type 1 diabetes patients visiting a tertiary care hospital of Nepal: A descriptive phenomenological study
Source: PLOS Glob Public Health. 2026 Jan 13;6(1):e0005810. doi: 10.1371/journal.pgph.0005810 (PMC12798998; doi:10.1371/journal.pgph.0005810)
Supplement: S4 Appendix — (DOCX) [file pgph.0005810.s005.docx]

Interview 1

The participant is a 15-year-old boy diagnosed with Type 1 diabetes three years ago, he went for treatment after experiencing severe symptoms like fatigue, thirst, and frequent urination. He was admitted to the intensive care unit at the hospital. Initially he was scared and anxious about the condition, he learned to manage his health through the support from his family and healthcare staff. He is self-administrating insulin twice a day and monitoring his glucose levels regularly. He follows a strict diet and remains physically active through daily chores. His mother has become more caring since the diagnosis. He missed his check-ups and had no access to online classes during COVID-19. He has experienced changes in his energy levels and eating habits after being diagnosed with Type 1 diabetes. Experiences related to occasional temptations to eat restricted foods when with friends shared by him. Satisfaction with the treatments shared. He is open to sharing his experience with others if given the chance.

Interview 2

The participant is a 21years old student who was diagnosed with Type 1 diabetes around 11 years back. She experienced symptoms like thinness and ants near the toilet. She was admitted to the ICU of the hospital. Initially she and her family were shocked and anxious about the diagnosis but with time and support from doctors, nurses, and especially her mother, she learned to manage her condition. She self-administers her insulin independently, adjusts dosages and monitors her blood glucose. Insulin management around her college timing and travel remains a challenge, especially maintaining proper storage and timing. Significant dietary modification reported. Despite the college timing and fatigue, she tries to do some exercise. She faces some lifestyle restrictions, such as limited travel and concerns about participating in sports due to fear of hypoglycemia. She is worried about future complications and her marriage prospects. The COVID-19 pandemic disrupted her follow-ups, caused concerns around vaccine safety and insulin availability. She finds her healthcare providers encouraging and helpful. Overall, she has adapted well and remains resilient in managing her condition.

Interview 3

The participant is 20 years old diagnosed with Type 1 diabetes 9 years back after fainting and being admitted to the ICU. He became aware of the chronic and lifelong nature of the condition only four years ago when he learned he would require insulin for life. The diagnosis caused significant emotional distress, including fear and anxiety. Over time, he adapted by learning from hospital staff and his mother. He adheres to dietary restrictions but struggles with limitations related to food choices, travel, and physical activities. The COVID-19 pandemic disrupted his routine care and access to insulin. Diabetes has profoundly affected his lifestyle, instilling a sense of restriction and uncertainty about the future. He reports financial strain on his family, increased caregiving responsibilities for his mother, and missed opportunities such as school trips due to teachers' concerns. He feels physically different from his peers and worries about future employment. Despite finding insulin injections and blood sugar fluctuations particularly challenging, he remains committed to following medical advice. However, he continues to worry about long-term complications and feels emotionally burdened by the need to depend on insulin for life.

Interview 4

The participant experienced severe symptoms leading to partial paralysis. Initially he was taken to traditional healers for nearly a year and was eventually admitted to the hospital, where he was diagnosed. At first, he felt alone and anxious, but seeing others with the same condition helped ease his emotional burden. With support from hospital staff and his younger sister he learned insulin administration. He now independently takes insulin, follows a balanced diet, and checks his glucose levels. COVID – 19 has disrupted his follow-ups. He is satisfied with the support from health workers, especially the motivation received through group activities. He finds insulin dosage adjustments manageable and doesn’t feel deprived compared to others of his age. Overall, he remains positive in his self-care and management of diabetes.

Interview 5

The participant was diagnosed after experiencing weakness and weight loss. Initially, afraid of injections, she gradually learned to administer insulin herself. She now manages her condition through regular insulin use, blood glucose monitoring, and moderate dietary control. However, she does not follow an exercise routine due to a demanding college schedule and reports difficulty maintaining stable blood sugar levels, particularly with episodes of hyperglycemia. She shared a significant missed opportunity when illness forced her to return home, preventing her from applying to a medical course. Despite challenges, especially accessing insulin and medical supplies during the COVID pandemic, she remains optimistic. Encouraged by seeing other children with similar conditions, and supported by her family, she maintains her health with confidence and resilience.

Interview 6Interview

The 16-year-old participant recounted his life with Type 1 diabetes since the age of diagnosis at 9 years old. He recalled experiencing symptoms like frequent urination and lethargy. He did not know how severe the illness was at first; he learned to cope with it slowly over time through instructions from doctors and encouragement from his mom. He takes care of himself nowadays through self-administering insulin, blood glucose monitoring, dietary caution, and frequent check-ups.
The participant exhibited trust in his self-care activities and satisfaction with health care services and school support. He practices informal exercise daily. He reported the hypoglycemic attack experience during a class 7 examination. He is optimistic despite having diabetes, an active sportsperson. He also surfs the internet to learn more about diabetes.

Interview 7

The 24-year-old participant has been managing Type 1 diabetes since he was diagnosed at the age of 10 years. He felt worried and alone with his condition initially but was reassured after meeting others who had the same condition through hospital follow-ups. He gradually learned through health professionals' advice and peer support about insulin dosage, diet control, and self-management. He now manages his diabetes on his own by taking regular insulin injections, timely meals, and glucose checks. He relies on the hospital for guidance and information and has also developed a support system via patient programs. While he suffered a severe hypoglycemic attack seven months ago, his condition has otherwise been stable. He took extra precautions during the COVID-19 lockdowns and had continuous work with no issues managing medicine and follow-ups. His primary concern is the upcoming termination of free diabetic care at the age of 25 and would prefer an extension. Overall, he is optimistic, independent, and well-adjusted to his diabetes.

Interview 9

The participant had initially struggled to understand the disease and had basically relied on her mother for advice and support, such as how to give insulin. Over time, upon the advice of doctors her mother began managing the condition independently. Her daily life now consists of insulin, controlled diet, exercise, and observation of blood sugar levels. She shared that the most difficult aspect is dealing with dietary restrictions, especially during parties or school. She also complained about adjusting insulin doses, particularly the afternoon dose when she is at school.
She talked about the social and emotional impact of her illness, for instance, frustration caused by uncontrollable variation of her blood sugar, missing school trips and after-school events, and limitations imposed upon her by her family due to health reasons. While she wished to be a doctor, now she is willing to be a graphic designer. The COVID-19 pandemic also limited her activities and studies, as her family took extra precautions. Overall, she is hopeful, but her diabetes continues to govern her lifestyle, decisions, and future life.

Interview 10

The participant was diagnosed with Type 1 diabetes after presenting with dry mouth and unexplained weight loss. He was initially frightened and upset but later comforted by support groups and subsequent hospital revisits. Despite being put on insulin, he later stopped taking it due to pain, scarring, and social inappropriateness. Without seeking the opinion of a doctor, he started taking oral tablets and Ayurveda medicine, which he claims control sugar. He follows a controlled diet, does physical exercise by going to the gym, and does not consume sweet food. Although conscious of long-term complications, he monitors his kidneys and eyes. He was worried about transmitting diabetes to his daughter. When he fell ill during the COVID-19 pandemic, he believed that he had contracted the virus but did not take tests, treating himself at home with rest and a well-balanced diet. Though he no longer goes to diabetes support groups, he emphasized self-awareness and taking charge of the disease.

Interview 11

The interview presents the experience of a participant who has been living with Type 1 diabetes since the age of three. She manages her condition with regular insulin injections, careful dietary choices, and moderate exercise. Although she has developed a strong routine for managing her diabetes, she struggles with night-time hypoglycemia and occasional unexplained spikes in blood sugar, which cause anxiety. Her mother plays an active role in her care, adapting their daily life to support her needs. The girl feels uncertain about her future due to the unpredictable nature of her condition, which has led her to reconsider her earlier ambition to become a doctor, fearing it might be too stressful. She stays informed through medical professionals and online resources and feels well-supported by her healthcare team. However, she is somewhat uncomfortable with injecting insulin in public, reflecting a sense of self-consciousness. The COVID-19 pandemic intensified her family's concerns, especially during times when she had to use a generic insulin due to supply issues. Despite these challenges, she maintains a hopeful outlook and continues to adapt to life with diabetes, demonstrating resilience and awareness of her condition.

Interview 12

The participant was diagnosed with Type 1 diabetes at a young age. She has only formal education up to grade 5 and does not engage in any occupation. She stays at home with her family. Her diagnosis came unexpectedly during a hospital visit initially for jaundice, later confirmed as diabetes, leading to hospitalization. She recalls learning to self-administer insulin through encouragement from hospital staff and gradually adapted to managing her condition despite the lack of consistent access to tools like glucometer strips. Emotional responses to her diagnosis included fear and confusion, but seeing others with the same condition helped her cope. Her follow-ups and blood sugar tests have been affected due to COVID-19 and lack of supplies. She did not continue her studies after grade 5, most likely due to her diagnosis. While her mother remains deeply concerned, she feels supported by her family. She remains uncertain about taking the COVID-19 vaccine but is open to further consultation.

Interview 13

The interviewee is a participant who was diagnosed with Type 1 diabetes at the age of 10 months. His diabetes has required ongoing care, including taking insulin regularly, dietary management, and blood tests such as HbA1c. He came to understand his condition over time, with the capacity to administer insulin independently at the age of 10. Despite initial fear and frustration, he gained confidence after undergoing training in hospitals and after getting support from others who have gone through the same experience. Despite difficulty, he manages his glycemic level through intensive food choices, regular blood glucose monitoring, and daily walking. Diabetes has significantly affected his life and that of his family. His parents made many sacrifices, including relocating to another location for improved care. He is afraid of the long-term effects of the disease on his organs, career, and relationships, and is especially scared of social stigma and future limitations. He is uncomfortable sometimes injecting insulin in public or disclosing his condition. COVID-19 caused problems in obtaining treatment. Despite the obstacles, he has a positive attitude, values the help of medical personnel

Interview 14

The participant shared her deeply personal journey of living with Type 1 diabetes since childhood. Diagnosed at the age of 6 in her village during her school exams, she initially experienced symptoms like frequent urination, excessive thirst, and weight loss, which were misinterpreted culturally and treated by a shaman before medical intervention confirmed the diagnosis. Despite early challenges, she learned to inject insulin herself and took charge of her condition with a sense of determination and maturity, even at a young age. Throughout her adolescence and young adulthood, she faced setbacks in education and career aspirations due to the condition like missing exams, had to leave career of her choice, and being discouraged from traveling or working outside the valley due to family concerns over her health. Despite these limitations, she maintains strong self-management practices, including strict diet control, regular sugar monitoring, and consistent insulin use. She identifies hypoglycemia, especially nighttime episodes, as her biggest ongoing challenge, expressing concern about being a burden on her family. She advocates for greater awareness in rural Nepal, citing harmful stigma.

Interview 15

The participant has been living with Type 1 diabetes for nearly eight years, having been diagnosed after collapsing at work and being hospitalized in an ICU. With only up to 6th grade education and working long hours in his job, he struggles to maintain consistent diabetes management due to time constraints and limited awareness. Though he injects insulin twice daily and attends check-ups every 2–3 months, he finds it difficult to consistently manage diet and avoid social discomfort when injecting insulin in front of others. He faces emotional distress, including fear, anxiety about future complications, and feelings of social isolation. He also shares the encounter with the lack of knowledge in society about the disease. His family, especially his parents, are supportive and concerned, although he lives apart from them. Financial strain, especially during COVID lockdowns, added further stress. Despite challenges, he has adapted over time with guidance from doctors and pharmacists, though he worries about missed opportunities, work limitations, and long-term impacts of the disease.

Interview 16

The participant was diagnosed at age 9 after a severe episode and led her to hospitalization. Initially unaware of the disease, her family took her to a shaman before eventually seeking medical care. Over time, she learned to manage her condition independently, including insulin administration, as her mother was initially too scared to help. She maintains regular follow-ups and has a stable HbA1C level, though she admits to being less consistent with exercise and occasionally indulges in non-recommended foods. While her condition initially led to social isolation and financial stress on her family, she now feels accepted and well-supported, though she missed out on a scholarship opportunity abroad due to her diabetic status. She remains concerned about future limitations, particularly the lifelong dependency on insulin, but generally feels positive and satisfied with her medical care and support system.

Interview 17

The participant has lived with Type 1 diabetes since a very early age, diagnosed at just 1.5 years old after a serious episode that led to a coma and hospitalization. Over the years, he gradually came to understand his condition and now manages it independently, including insulin administration and diet regulation, although maintaining strict routines is challenging due to his busy schedule. He has experienced severe hypoglycemia in the past but now manages better through awareness and timely food intake. While he feels emotionally resilient today, he occasionally reflects on missed opportunities and worries about passing the condition to future children. Support from family, especially in avoiding sweets at home, and positive experiences with healthcare professionals have helped him cope. Although he faced early hospitalizations and dietary restrictions, he maintains a proactive and informed approach to his condition.

Interview 19

The participant has been living with Type 1 diabetes since the age of 12years, facing a long and emotionally difficult diagnostic journey that began with misdiagnoses and traditional healing practices before proper treatment. Initially overwhelmed by fear and the lifelong requirement of insulin injections, she gradually adapted to her condition with the support of healthcare professionals and a peer group. She now manages her diabetes through regular insulin use, diet control, exercise, and routine checkups. Though she leads a largely independent life, she still faces social stigma, emotional strain, and challenges related to travel, social eating, and long-term complications. Family support has been strong but also comes with overprotection, especially regarding marriage prospects. Her condition once affected her academic path, forcing her to change majors. Despite managing well, she remains cautious about the future and potential complications, particularly those affecting kidneys and eyes, while also navigating restrictions and misconceptions from society.

Interview 20

The participant was diagnosed unexpectedly during a routine checkup after experiencing frequent urination. Despite having a significantly high blood sugar level (around 820), he remained symptom-free and was admitted to the hospital for 10–12 days. Initially shocked and fearful about lifelong insulin use, he gradually accepted his condition through self-learning, peer support, and observation of other patients. Since then, he has followed a structured routine involving insulin administration, dietary control, regular exercise, and consistent follow-ups, maintaining an HbA1c below 6.8. While he occasionally feels burdened by the lifelong nature of insulin use and fears complications during old age, he has not faced major disruptions in his work or personal life. Financial costs, particularly for tests and insulin, are a concern, but he handles them as a necessary part of staying healthy.

Interview 22

The participant was diagnosed after experiencing common symptoms like frequent urination, excessive thirst, and weight loss. Initially treated with medication, his condition worsened, and he was admitted to the ICU, where he began insulin therapy. Since then, he has been on insulin, maintaining stable HbA1c levels. Despite initial anxiety, he adapted to his condition through self-education via hospital classes and extensive internet research. He actively manages his health with insulin, a routine lifestyle, diet awareness, and exercise, while also mentoring others with diabetes and advocating for a support organization for Type 1 patients. Though financially strained by the high cost of insulin and occasionally fails to follow with diet during social events, he has avoided severe hypoglycemia and remains functional and optimistic. He hides his condition socially and professionally to avoid stigma, and while he acknowledges long-term complications, he feels empowered and focused on growing his career .

Top of Form

Bottom of Form

Top of Form
