# Supplementary material for: Lived experiences of Type 1 diabetes patients visiting a tertiary care hospital of Nepal: A descriptive phenomenological study
Source: PLOS Glob Public Health. 2026 Jan 13;6(1):e0005810. doi: 10.1371/journal.pgph.0005810 (PMC12798998; doi:10.1371/journal.pgph.0005810)
Supplement: S1 File — (DOCX) [file pgph.0005810.s006.docx]

**I : Interviewer P: Participant**

I: How many years have it been since you got diagnosed with Type1 diabetes?

P: It’s been about 17 years now; I got diagnosed in 2061. Since now it is the year 2077, it’s been 17 years.

I: How old were you when it was diagnosed?

P: I was about 13-14 years; I think 14 years old.

I: So, you were about 14 years old. Who else besides you in your family have this disease?

P: Yes... (Pause) My older brother.

I: Your older brother has it?

P: Yes.

I: Ok, so you got diagnosed after your brother. Was there anyone else who got diagnosed?

P: My brother got diagnosed first. He was diagnosed two years ahead of me, I think. I was diagnosed in 2061 while he was diagnosed at around 2058/59.

I: You were diagnosed in 2061, right? There was no one else who was diagnosed before him, right. Have you been doing the HbA1c test?

P: Yes, I have been doing the test, in P…. But currently I haven’t been able to visit the P.. Hospital due to lockdown and all. So now I am doing the test in …….., and Kathmandu is far away too.

I: Ok.

P: I have not been able to go to the hospital in Kathmandu. (Laugh) Although we must do this test every three months.

I: (smile ...), It is the same wherever you do it.

P: In the recent period when I was taking the prescribed amount of insulin from the P… Hospital, my sugar level continued to be on the lower side, so I reduced the amount of insulin. But after I reduced it, it became high. I checked in here to find out the level and it showed in the higher range.

I: How much was it?

P: I did it this month, it was about 9 I think, so it was high and after that, I injected the amount as initially prescribed by the doctor and again it is consistently in the lower range now.

I: So, you have to adjust the dose then, have you consulted the doctor?

P: Yes, I went to the health facility here, but they said they could not help and told us to go to Kathmandu, P….. Hospital.

I: Yes, since the doctor you were consulting first will know your medical history, you can visit him once the COVID becomes less severe.

P: We are a little anxious and worried due to COVID taking risks as we are in increased susceptibility, so not being able to go for the follow-up.

I: Ok …. ……, how did you find out that you have Type 1 diabetes? Please share your experience.

P: I found out I have Type 1 diabetes in P…. Hospital. I was admitted to the hospital for around 10 days.

I: What happened to you then?

P: My weight kept on decreasing then. I kept feeling thirsty, had to pee a lot, felt hungry, felt sleepy all the time, and like these kinds of things. I knew a sister from P… hospital, and she told me it could be diabetes and took me to the hospital with her for a checkup and there I was diagnosed with diabetes. At that time my blood sugar was 500, it was fasting I guess and then the doctor told me to get admitted immediately. I was admitted to the hospital for about 1 week.

I: Were you admitted to the general ward, or had you been admitted to the ICU?

P: I was admitted to the General ward. I went for a check-up in room number … and from there they sent me to the ward and gave me a bed.

I: How did you feel when you found out you have Type 1 diabetes? Please share your experience.

P: I did not understand.

I: When you went to the hospital and they said, you have Type 1 diabetes, how did you feel then? Please share your experience.

P: Since my brother was already diagnosed at that time, I did feel uncomfortable. I thought that is how it is and consoled myself. (smile)

I: (Smile)....... At that time because your brother already had it you were not worried when you had it, please share your experience?

P: I was not anxious; it was not something I could control.

I: Who was there to accompany you to the hospital?

P: I was the only one in the hospital then.

I: You were alone when you were admitted to the hospital?

P: I was working and studying in Kathmandu; I went to the hospital for a checkup and then got admitted to the hospital. My brothers and sisters were also in Kathmandu then. After the doctor told me to get admitted, I called and told them. They said to go ahead, and they will come to the hospital later. They only came to the hospital later in the evening.

I: After you went alone, your siblings came in later to accompany you?

P: In the evening, my siblings came to accompany me as my attendants.

I: After that, have you been managing diabetes on your own? What do you do for diabetes management? Please share your experiences related to diabetes self-care management.
P: Now, there is a diet restriction, which I have not done much. When I go out with my friends I drink cold drinks as well. I don’t smoke or drink alcohol. I do drink cold drinks, sometimes when my friends offer like one glass. I eat normally; whatever others eat. Things have not become that difficult (smile).

I: Besides diet, you must have also learned how to take insulin and take it regularly?

P: Yes, I have been taking insulin injections regularly.

I: Have you also been doing routine blood sugar tests on your own?

P: Yes, I do that as well. Routinely, I used to do my blood sugar test at home but since the kit is finished and it is not available here, I have not been able to do that now. I have to go to Kathmandu to find them.

I: You used to do that before; besides that, you were probably told to go for follow-up and maybe other things too, what were you told then? Please share your experiences.

P: Oh….. at that time (pause) I was told to control my diet. I was given insulin; I was taught how to take insulin. I was also told what I should eat, about portion sizes in the list. I was given the list for that.

I: Who taught you these things then?

P: At that time, I was taught by the two doctors. They both looked after me. I am still under their follow-ups when I come to Kathmandu.

I: Who learned how to take insulin first you or your sibling?

P: Ok…. At first, when I was in the hospital I was taught by the doctors, and during my hospital stay I had to learn how to take it because eventually, I had to take it on my own.

I: Ok. After that, you took it on your own?

I: Since for diabetes management you have to do blood tests, take insulin, adjust the dosage of taking insulin, etc. what aspect among these do you find challenging to do as per the doctor’s advice? Which of them are difficult to comply with?

P: We are told we have Type 1 diabetes and there are Type 1 diabetes, Type 2 diabetes, and Type 3 diabetes, what are those? I have not understood these things much to date. For Type 1 diabetes, I wish we could take tablets instead of insulin. Regarding food, it is difficult to manage as per advice. I wish I could just have medicine.

I: You said it is difficult for you to take insulin and adjust the dosage of insulin. Please share your experience in this.

P: Regarding the dosage, if it is high, you lower the dosage and if the sugar level is low… (he realized his mistake and smiled) Oh, if the level is high, you increase the dosage, and if low you lower the dosage. That is how I am doing it.

I: You also said it is difficult for you to go for follow-up. Please share your experience on this.

P: It is difficult to come.

I: Yes, since you must leave work in R………. for this, so…..

I: How is that?

P: Regarding follow-ups, because we live in the village it is difficult to come from time to time. Staying in hotels is not feasible. Moreover, in the hospital for a blood test, we must go one day ahead. We leave the village one day ahead and we must stay at our relative's place. We must go the next day to give blood, go for a checkup, then the following day meet the doctor. Then only our turn will come on the same day. All these things make it challenging for us to go to the hospital for checkups timely as advised by the doctor. It would have been easier if we were living in Kathmandu or any one of my close relatives was there. Since we live in the village and so do my siblings, it is uncomfortable to stay at relatives and neighbors’ place, so it’s been a long gap this time, about 2 years we have not gone there.

I: Ok. What sort of changes do you feel having Type1 diabetes has brought in your life?

P: What can I say about changes, it’s just I am not healthy as others. (Laughs) I am not as fit as others, and I wish I was. That is, it.

I: What sort of changes do you feel Type 1 diabetes has brought in the life of your family?

P: Regarding changes, we cannot travel and there aren’t many jobs in the village. That is, it.

I: What else, please share if there are any.

P: (laugh) That is it, in the village the financial situation is precarious, and I am unable to leave the house to improve my financial situation. I want to be financially secure as well as create financial stability for the family. All these financial difficulties have left us wondering how we will handle our medical needs, where we will acquire the medicine, and what we will do in the future; we are concerned about such things.

I: So, you and your family have experienced financial problems?

P: Can Type 1 diabetes patients go abroad?

I: Pardon …..

P: I wanted to ask the doctors as well.

I: Ok.

P: Do they send/allow Type 1 diabetes to go out of the country?

I: That depends …...

P: Ok.

I: That depends on the rules and policies of a country, what their requirements are. Otherwise, it is maintaining diet, taking insulin. It is just like any other disease where it does not matter if you can take good care of yourself, I can say this much on this now. You have to consult the doctor about this as he can provide you with a detailed suggestion.

P: Oh, yes.

I: You have to work long hours, within that you have to look into break time, dietary arrangements, will they give you time, there is a matter of rest as well – you have to look into all these aspects.

P: Oh, ok ok.

I: Have you been looking into foreign employment?

P: That is something I have not consulted the doctor with.

I: Ok.

P: Now, my family also says to try for foreign employment since there is not much of opportunities in our country. You don’t get much work; you need a certain education level as well. I have only studied till class 12, so there is a mentality that maybe something will happen if I could go into foreign employment.

I: Ok.

P: I must consult with the doctor on this matter. I also wanted to know about medical insurance. I wonder whether all these are included in it or not

I: That is something I will have to look into.

P: Ok.

I: For health insurance, there are criteria based on common medicine, I need to check up on that.

P: Ok.

I: I will look upon these and inform you later. One thing I can suggest to you is to go for the follow-up and consult the doctor on these matters too as you have priory said that your blood sugar level is continuously in the lower range. If it’s not possible to come to Kathmandu, then you can also do the consultation there. Is there a hospital in …….. having these facilities?

P: There are hospitals in my place, but they do not provide you with treatment as per expectation. They admit you here even if you have a fever and then refer you to somewhere else 4-5 days later. They diagnose you with one illness and then refer you to Kathmandu where you get diagnosed with another. Hence, I have trust issues with the place here. But still, I did my check-up here 2-3 times as it was required. During a checkup, they told me that I should go to Kathmandu rather than come there as they cannot help .

I: Maybe they said that because they do not have a doctor who is specialized in this field, as for this illness you need a specialized doctor?

P: So, I have a concept like why to go for a check-up in the village (laughs).

I: That is fine, I will enquire about that. You can call your doctor too.

P: Yea, sure.

I: Do you ever worry that due toType1 diabetes you might face problems in the future? What things about your illness concerns you? Please share your experience.

P: I am not worried about it much, what I do worry about is, till the end of my life I have to take this medicine, I have been managing it now as I am earning, but how will I manage in the later future.

I: You are worried about how you will manage the money for the medicine?

P: Yes, in the future I will manage till I can work but what about when I can’t, I am worried about that.

I: Besides that, do you worry that you might get any other illness because of this?

P: Yes, I do worry. I am concerned about the long-term effect of taking this medicine, would it cause many problems. The main problem with us diabetic patients is the effect on kidneys and eyes, I do worry what if it affects any one of my kidneys or eyes, what will I do then?

I: Are you married or unmarried?

P: I should say I am married now .......(laughs)

I: Have you experienced any concerns related to your offspring having this disease? Do you have kids?

P: We have not even planned for the baby yet. My partner and I are not even able to properly settle down till now. If we have children while I am not well myself, who will look after my children in the future? What if we have children and me being the breadwinner something happens to me, then it will be horrible. So, we decided that we are not going to have kids for now.

I: Do you worry that your child might also get this disease?

P: It might, it might not, my brother has 2 kids one is 17 years old the other is 12-13 years old, both of them have not shown signs of the disease till now, not sure if they might in the future.

I: Because of diabetes you have been admitted to the hospital once, have you been admitted to the hospital again after that?

P: I have not had to be admitted to the hospital after that.

I: From time to time, you go to the hospital in ……… and …., in those visits what is your experience with the hospital staffs? How is their behavior towards you? Please share your experience.

P: It is fine till now. In P…, there is an organization that provides a package to those who are under 25 years, where they provide most of the medicines for free. They also run many programs on Type 1 diabetes. I was also benefitted by this, I took the free services of medicine for around 10-12 years, at that time Dr, S… was looking after us. Dr….. also looks after us well. They are nice when we visit the hospital.

I: They are good right, how satisfied are you with the treatment you are receiving?

P: The treatment is good. I also take references from the net sometimes. Type 1 diabetes is a dangerous one, you must take insulin. I take it as there are benefits of taking insulin too*.* It is fine. It is fine, even if I was unsatisfied there is nothing I can do. I have to be satisfied, don’t I? (laughs)

I: You shared that you were unable to go abroad, besides that what sort of other activities do you feel you are prevented from participating in because of this illness? Please share your experience.

P: I have not tried many jobs. I cannot say that it is because of diabetes, it’s just if I could go abroad to work it could help us financially, that is it nothing else.

I: Sometimes you have to travel outside with friends, and in terms of meals how restricted do you feel?

P: I haven’t drunk alcohol since before, because of that I don’t care about that. Sometimes my friends do push me to drink cold drinks, and I take it.

I: Ok.

P: In terms of traveling, I do go out when I want to. It is a little problematic when we go to our relative's place because I must take medicine (insulin) and because of that I feel it is a little difficult and uncomfortable. It would have been easier if it was just a tablet, no one would know (laughs).

I: You feel uncomfortable, right. And?

P: And since I am quite young, about 30 years old, I don’t find it comfortable to disclose my diabetic status to my relatives. If they know then they will start buzzing around like oh you got this disease at such a young age, oh you have this problem at such a young age and all, due to all these causes I don’t find it comfortable to tell them.

I: You shared that you feel uncomfortable using the injection in front of your friends and relatives. Have you experienced any kinds of discrimination from friends or family because of being a Type 1 diabetic person?

P: Nothing like that, they have not said anything directly to me. (Laughs)

I: Has your brother shared with relatives that he has Type 1 diabetes?

P: My brother?

I: Yes.

P: Everyone in my family knows about this.

I: ok.

P: Regarding my wife’s family and relatives, I feel uncomfortable. And, when I have to visit some of my relatives like my sister’s family it gets uncomfortable then.

I: Ok.....

P: We have not openly shared that we have this, so we feel uncomfortable; even if we have and some of them know but still it is uncomfortable to take the injection in front of others.

I: You feel uncomfortable right?

P: Yes

I: Ok. Since now it is COVID time, please share your experience related to living in this pandemic.

P: We heard it in the news and the doctors told us that diabetes patients might face difficulty repeatedly in the news. Concerning how to manage, how to get the medicine was present. It was difficult during the lockdown. Till then insulin was not available in ………. Then I requested saying I must take this medicine regularly, I will buy it. Only then do they start procuring it and making it available in their pharmacy. I go there and order first and then they make it available as per my order.

I: Ok.

P: During the first wave, it was difficult for us. No public vehicles were running, and we do not own any. If I had my bike, then I could have made a pass to get the medicines, and it would have been easier. There in that condition, if I had to reserve a vehicle it would cost about Rs.5000 for going to Kathmandu only and Rs.10000 for two ways. It was difficult. I knew an ambulance driver and he helped to get the medicine after I explained the situation*.*

I: Ok

P: They got us the medicine. I have not gotten covid yet, they say if I get it, it will be dangerous.

I: Have you taken the covid vaccine, sir?

P: There is no vaccine here. I heard it was available for people above 35 years old. We have not gotten it yet.

I: Once it gets there, do get the injection and be safe.

P: Can we Type1diabetes patients take the vaccine?

I: Are you talking about the covid vaccine?

P: Yes.

I: Yes, you can.

P: I have heard that even a healthy person gets sick after getting the vaccine is it right? I worry that we sick people might get sicker because of this vaccine. Many of the people here got sick after getting the vaccine. I was worried that since I am already sick if I get vaccinated, I might be paralyzed, and it might cause more problems*.*

I: (Laugh) Some people will be affected. …, there is a chance the vaccine could affect anybody, not just diabetic persons but it is not the issue that because you have diabetes you cannot take the vaccine. It is more beneficial to you if you take the vaccine.

P: Ok

I: During covid you told me that it was difficult to get the insulin, besides that you also said that it was difficult to get the strips for the blood test. Please share your experience with it.

P: You cannot get the strips here till now too.

I: Ok.

P: Have to go to Kathmandu for that. One month ago, I went to the medical here for a blood sugar test and it showed high, it was about 110. I am planning my follow-up at P…. Hospital.

I: During covid did you continue your painting work, or did you stop for a little time, resume it after the lockdown or after the situation become a little normal? Do you have experiences related to this?

P: Regarding work because this is a small market area it is difficult. Even during the lockdown, I worked a little secretly, not much. (Laugh)

I: Please share.

P: During covid, I worked a little not a lot; it did not get too difficult. It is a little difficult to find work as it is a small area.

I: If there was no covid then you could have come to Kathmandu as well, maybe worked here.

P: If the covid situation was completely resolved I could have gone to Kathmandu. Although, I had stayed in Kathmandu for 8-10 years before, and as it was difficult to get work, I returned to the village.

I: Did you not get to work because you had diabetes or were there other reasons?

P: Me returning to the village?

I: You said you returned to your village because you could not find work.

P: Yes.

I: Did you not get to work because you had diabetes or some other reason?

P: It was not like that, at that time I was studying and working, and it caused problems. I could not concentrate on my studies if I worked.

I: Ok.

P: As I couldn’t get proper work in Kathmandu, I returned to my village. It had been about 8-10 years since my return to my village. I returned on 2068/69. After I returned to the village 1-2 years later, I could not get along, so I moved to the town area of my village learned how to paint and do it now.

I: It was a nice conversation, is there anything you want to share further?

P: I wanted to ask you, they say I have Type 1 diabetes, how do you differentiate between Type 1,2, or 3?

I: Regarding Type 1 and Type 2. In Type 2 the body produces insulin but it is insufficient so you can take medicine to fix that. In Type 1 the pancreas producing insulin becomes defective and it cannot produce insulin so in that case, you must take insulin.

P: We must take insulin continuously?

I: As your body is not producing insulin you must take it from external measure. There is no alternative to injection yet.

P: In the future, it might be possible through science.

I: To date, they have not been able to find a solution; we must take an injection but who knows in the future.

P: There are advertisements on ways to cure diabetes through Ayurveda, they say this cures diabetes.

I: That is for Type 2 whereby maintaining your diet you can control it but for Type 1, insulin is the only option.

P: You cannot control even by maintaining a diet?

I: By controlling and maintaining your diet you can take a low dosage of insulin, if you can control your diet insulin dose might not be increased further but you cannot stop taking the insulin. Once you are diagnosed you have to take it.

P: Ok.

I: Is there anything else?

P: Oh, no.

I: Ok thank you for giving me your time.

P: Ok
