# Supplementary material for: Lived experiences of Type 1 diabetes patients visiting a tertiary care hospital of Nepal: A descriptive phenomenological study
Source: PLOS Glob Public Health. 2026 Jan 13;6(1):e0005810. doi: 10.1371/journal.pgph.0005810 (PMC12798998; doi:10.1371/journal.pgph.0005810)
Supplement: S2 File — (DOCX) [file pgph.0005810.s007.docx]

**I= Interviewer P= Participant**

I: How many years has it been since you got diagnosed with Type 1 diabetes?

P: It was 2067 when I first went to Patan Hospital and got admitted. We did not know then my sugar level was very high. Although we knew few symptoms of diabetes then but we thought about how it could happen in childhood. I thought it was due to weakness, so I drank a lot of juice, and the sugar level further increased and reached up to 700. I was already unconscious by the time I was taken to the hospital. And I was admitted there. I was diagnosed and I have had this disease from then.

I: Do you know if anyone from your family has Type 1 diabetes?

P: Yes, my mom and sister have diabetes but the other one not Type 1.

I: Have you been doing the HbA1c test?

P: Yes.

I: Ok.

P: Yes I do it every 6/6 months or every 3 months.

I: Do you remember the last value?

P: I think it was 7.8 %.

I: Ok.

P: It was 2/3 months ago.

I: You shared that you were admitted to Patan Hospital, what were the symptoms at that time?

P: I had to go to the bathroom a lot and I was very thirsty, but the most difficult thing was I could not walk, I was feeling that weak.

I: So you did not go immediately.

P: Yes, I even fell from the ladder once. And at that time I felt that maybe my body hurts because of that.

I: How long did you feel that way? How long did you feel the symptoms?

P: I went for a checkup at a local health facility after about a week, but the urine test was remaining. I passed out as soon as I gave my urine to the lab for testing and was taken to the hospital immediately, I did not know then as I was unconscious.

I: Were you admitted in the general ward or in the I.C.U.?

P: I was kept in I.C.U.

I: So, it got really difficult for you.

P: Yes, it was very difficult.

I: How did you feel when you found out that you have Type1 diabetes?

P: I felt very bad. At such a young age, I was diagnosed with such a disease where I had to follow an instructed diet and all the other things as I had just given SLC and was studying +2. I felt terrible at that time as I couldn’t eat like my friends and also there were several things to be considered. As I started to visit the Patan Hospital, I met many people with the same diagnosis, and we became friends. I even came across a 2-month-old baby with diabetes. And after that, I felt it was not a big disease to stress about and take tension. As time passed by after 1-2 years I felt this was normal.

I: At first how did you feel?

P: At first I was horrified. I felt like what kind of illness is this where I have to take injections daily? My sister used to give me injections, but once the doctor told me that I needed to learn how to do it myself, I learned it and did it.

I: At that time did you cry out of anxiousness?

P: I used to cry a lot. When I saw food and I couldn’t have them I used to cry a lot. Especially the sweet foods, I craved eating sweets then.

I: So slowly you started to learn right, making friends with the other people with the same diagnosis helped too?

P: Yes, I made a lot of friends. Looking at small kids, I felt grateful that at least at that age I could eat anything I wanted, I felt bad for the children. There were kids who couldn’t even speak. Now I think about the pain that small children’s parents must be going through. As they cannot eat otherwise so the small children just kept on making excuses of hypoglycemia to their parents so that they were given chocolates to eat. And when I see that I felt grateful that I got diagnosed at a more mature age.

I: Ok, and what do you do for diabetes management? Please share your experience.

P: Sorry, what did you say?

I: You have been managing diabetes on your own at home; please share your experience on this.

P: Most of the time my sugar level is on the lower side. For the initial two years, I completely modified my diet. I used to have only ‘*roti’ and ‘dhidho.’* By following such kind of diet my blood sugar started to remain consistently on the lower side so I thought it’s not working then I started to have normal rice. Now also whenever I consume ‘roti’ or ‘dhido’ my sugar level drops down very fast so I have normal rice but in lesser quantity.

I: What about sugary products and potatoes are you avoiding them or having them too?

P: I have been strictly avoiding sugary products and potatoes. Other than them there are not many items that I avoid. As my sugar level is on the lower side I don’t avoid many of the items and consume them in balance.

I: Have you brought some changes in your food patterns, besides those are there any more things which you are doing for your diabetes management?

P: Before I used to go for a morning walk sometimes, but now I have not been able to due to lack of time. Priorly I used to go for walks and even had attended some yoga sessions but now I am not doing them as I have no time. And also I don’t see there necessitates as my sugar level is on the lower side and further lowering can cause a problem .

I: Yes time factors for you as you have said you are busy with your household works, business works, and your studies, wow!

P: Not to forget I also have a small kid to look after.

I: oh kid too. And besides all these what about the regular blood sugar monitoring at home are you doing it.

P: Yes, I have the machine to check blood sugar at home besides checking it regularly; I can also tell about blood sugar levels whether it is high or low based on how I am feeling. You can also tell from the eyes as well. And according to all this, I have my meals.

I: Do you go for a checkup from time to time? You also mentioned the checkups before, do you go for regular checkups?

P: Yes, I go for the follow-up visits too

I: How many times do you take the insulin?

P: In the morning, as the blood sugar level rises it's 24 units and 18 units in the evening. I am using the 30/70 combination.

I: So you have been taking insulin, maintaining your diet, going for follow up and also doing exercise?

P: Yes.

I: Do you have experience of following a routine? Like the experience of arranging the time for insulin and food etc?

P: I had it initially but not anymore as it is difficult to manage time, it does not go according to plan.

I: It must have been difficult due to college timing.

P: Yes, Not only the college timing, there is the timing of shop as well. Sometimes you just have to go from the morning, you cannot be sure. I have not been able to eat in balance these days.

I: So you have not maintained your timetable?

P: Yes, these few days, the timetable is not fixed. I could not, there is no time.

I: Where did you learn all the things you have been doing for diabetes management?

P: Some people are senior to me in Patan Hospital.

I: Yes.

P: And where they share their experience, they have had this disease for a very long time, they had it since childhood, and there are 2-3 of them who are very much experienced. They shared their experiences in the get-togethers about what to do and how to do it. Besides that, the nurses used to take classes regarding diabetes. I do not remember their names; there was also a pharmacist who used to take the class. By all this, we all learned a lot. Our classes use to be on the first and last Tuesday of the month. There were foreigners also who used to give information in our classes. After taking that class we got to know what to do, how to do .

I: So you got information on diabetes from the hospital. Besides that, do you have experience of gathering information regarding this illness from anywhere else?

P: In terms of useful information for me, I look and search on YouTube a little. Besides that my family members also tell me what to eat and what not to. That is it besides hospital it is from the family and YouTube.

I: How has the diagnosis changed your life? Please share your experience.

P: There have not been many changes. I used to feel that way initially but now that I am used to it I feel this is a common disease. I feel now in Nepal there is no place where there are no diabetes patients. Isn’t it.

I: What sort of changes did you feel in the beginning?

P: What sort of changes were there?

I: You shared that there were changes in your food habits, besides that what else?

P: In that terms, it was a little difficult in traveling like going out for picnics, or night outs because we have to keep insulin in the freeze. Sometimes, there is no freeze, especially when going to the villages so difficult to manage. And also I found it full of hassles to carry insulin around while going out like that time I did not use to use pen insulin. As we were all provided with the insulin vial, syringes, and other items for free by the hospital up to the age of 25 years, I used to use that insulin at that time so a little bit uncomfortable . Now I use pen insulin so not much tension in maintaining temperature, I just simply carry it with me and just go to the bathroom take it and have my food.

I: Other than that do you have experiences of any extra precautions taken during travelings like not going out alone in the long-distance traveling due to the fear of hypoglycemia or anything like that?

P: Yes?

I: Now, for example, because of the fear of hypoglycemia you have not traveled alone for long distances or any extra precautions during traveling, do you have any such experiences?

P: Yes, Previously, my family would not allow me to travel to areas that were even remotely distant. My life just used to revolve around ……. Out of fear of my wellbeing, I was sent to the nearby college in ……. I just used to go to the nearby places but afterward, when I was able to take care of myself I slowly started to travel around a little.

I: Ok and you shared that you carry pen insulin with you while you travel, what about food, while traveling?

P: I eat foods I can eat. If I have to drink tea I have one without sugar, and I do slight compromise while eating. I eat the food items which I can have in lesser quantity. If I have to take tea then I go for tea without sugar, in this way I take meals in a compromised way during traveling

I: How has the diagnosis changed the life of your family members? Please share your experience.

P: According to my experience, they become more concerned and care a lot due to fear of wellbeing. As I was young they were always concerned if something happens to me. Not no major changes but in the initial period they also modified their diets and stop consuming things which I had to take precautions. They controlled the consumption of sugary items. My mother and sister did not consume food items before me which I had to take precautions; if they want to consume for example sugary items sometimes then they would consume them secretly. My mother was also diagnosed after my sister and I was diagnosed. She is no longer alive; she died. My younger sister was diagnosed with diabetes at one point, but she was able to manage it by changing her diet. My sister and brother have also entirely stopped eating sweet foods since then. They've also changed their eating habits. (smiles)

I: Besides that, you shared that your mother used to worry about you a lot? Please share your experience on this.

P: Yes, she used to scold me a lot on things I cannot eat. (laugh)

I: Do you feel this illness has added any sort of financial burden to you or your family?

P: In terms of financial hardship, there is an additional burden because insulin is costly. As a result, you'd have to spend a lot of money on medicine. I don't have my source of income; everything I do is from the money of business, so there's tension because I sometimes feel obligated to remain dependent (laughs). That's it; because I don't have employment, I have to rely on my husband for medicine.

I: So you feel there is a financial burden, right?

P: Yes.

I: How do you see your future?

P: Sorry I did not understand.

I: How do you see your future ma’am?

P: Yes ma’am, just a second I at the store, please continue.

I: What aspects of your diagnosis worry you the most? Please share your experience.

P: They say that as time passes and the duration of a diabetic patient's diagnosis lengthens, the patient's memory deteriorates. I'm starting to feel like I'm losing my memory.I'm not sure if I'm losing my memory or if it's just a weakness. When I have problems reading, I sometimes think it's because of memory loss. I am getting very forgetful; I just easily forget my lessons no matter how nicely I study. My other friends with the same diagnosis used to tell me that after sometimes the diabetic person starts to experience memory loss now I am feeling that I am going through all this. Priorly when they were saying I did not gave much attention now I am experiencing it.

I: It could be, do consult the doctor regarding this on your next follow-up. It could also be because you are so busy, as we can forget things when we are busy. Do consult the doctor on this topic too in your next follow-up

P: That is true.

I: Besides that just as you shared about memory loss, are there any other aspects of your diagnosis which concerns you a lot?

P: Yes, complications in main organs as well. In eyes, for example, I cannot see well from my one eye. My right eye is slightly weaker; I cannot read what is written on the opposite side. Also from my experience, I have found that when your sugar level is out of control then you have trouble seeing. If my sugar level is fine then I can see but if it is not I cannot see. Mainly the concern is related to the complication in kidneys, heart, and eyes. Among them, I am mostly concerned related to the probability of its impact on the Kidneys.

I: Besides that, are you also worried that in the future it might be seen in your children?

P: Yes, I am worried about it that is why since her birth from time to time we do the blood tests of our daughter, due to fear that whether it is present or it might happen. If not diagnosed in time, diabetes can cause a problem. Till now everything is fine, it is not seen.

I: Ok. And how do you see your future,…?

P: What to say I am not sure, right now. I feel like I won’t be able to do anything in the future.

I: Why is that ….?

P: I've studied a lot as I am now doing my master's level, even though I have recently joined after missing a lot of academic years. In the interim, I tried my hands on agribusiness, but it didn't work out as planned. I'm not completely healthy because I have diabetes, and I get sick from time to time. My biggest issue is a low blood sugar level. As a result of all of this, I have no idea what will happen in the future or how things will turn out.

I: Among the components of diabetes self-care management, which of them do you find challenging, or find it difficult to comply as per the doctor's instruction?

P: You mean challenging?

I: Yes, According to your experience which do you find most challenging to do?

P: Nothing like that.

I: Sometimes when you are sick, have you faced any difficulty in adjusting the insulin dosage?

P: I haven’t gotten that sick. I just manage it myself as I have many responsibilities. I manage it somehow I cannot just be sick. That is why even if I feel slightly sick like have a fever, I cannot sit still. I will manage myself and just work. I don’t have a habit of sleeping when I am not feeling well, I will just work and then I will feel better. Yes, that is why I have not had many problems, just had some complications during delivery.

I: What had happened then?

P: At that time my blood pressure suddenly raised and also I developed gastritis so despite already being in labor, I did not feel the pain. Due to high blood pressure, I was rushed to the hospital, while we reached there the placenta abruption had already occurred. I had to go under emergency operation and was kept on a ventilator. After 72 hours I regain my consciousness.

I: Oh, it was very difficult for you.

P: Yes, it was like that, the first time when I was diagnosed it took 45 hours, and this time during delivery it took 72 hours for me to be conscious. It was really difficult at that time.

I: And you have to go for a checkup from time to time to the hospital. How do you find the behavior of the hospital staff towards you? Please share your experiences related to the health care facilities and their staff.

P: That depends on their mood; it is difficult for them as well. Sometimes it happens, however that never happened in P…. Hospital since they prioritized all of us. Even for medicines, we are not required to wait in the queue. We could go to the doctor even if we bought the ticket at 10 a.m. We all know the people over there, so it's never been a problem. We merely refer to them as aunty or big brother, and because we have to see them regularly, we keep conversing with the staff and getting to know them in person. So never faced any problem.

I: Did they answer your queries?

P: Yes, very much

I: Are you satisfied with the treatment you are getting?

P: I am satisfied, it’s not bad.

I: Have you ever wished that there was some alternative for insulin?

P: I'm used to insulin; but, if I have to take tablets, it will be more difficult for me because I'll have to adhere to a much stricter diet. I have to eat outside things because I am constantly out of the house. That is why I believe insulin is beneficial to my health.

I: You should not also go for any alternatives; I just asked if whether you ever felt that way?

P: Oh, yes sometimes I do feel that I wish I could just take tablets but then I think I cannot avoid foods, I will have to eat outside. I feel insulin is better than tablets. That is why I think insulin is fine.

I: Have you ever experienced any form of deprivation from any form of activities or opportunities due to Type 1 diabetes?

P: Yes, initially at first I missed numerous opportunities.

I: Why? Because it was outside the valley or something?

P: Yes, my family did not use to send me anywhere, constantly they would say you are sick and that continued resulting in overprotection and over-care by the family. After when it come to job opportunities there too my family objected by saying, you will go for one day then get sick for 10 days, so I missed many opportunities. On top of that, I could not also study the course I wanted. These all things let me the feel of why?

I: Oh, there is a course you wanted to study but you couldn’t, ….?

P: Yes.

I: What was that course,…..?

P: That, I have my ….. uncle’s house in…., and I was not allowed to stay out of the valley not even there. I told them I would stay there and do some job but was not allowed. I was not allowed for the job here too. I was just told either you go for a government job or just to remain like that (laugh), doing that I could not do any jobs, I was not allowed, I was just told to study.

I: Besides that, you shared you were not allowed to go for any outings from school, right ma’am?

P: Yes, right after +2 I could not go anywhere, but during SLC, when I was in grade 9/10 since I was not diagnosed then I was able to go for a tour and go travel far. But now I have started going.

I: Have you faced any sort of discrimination or stigma related to your diagnosis? Please share your experience.

P: Yes, a little from my relatives and neighbors. They keep telling me diabetes is a huge illness and that I should not and cannot eat this and that, and also always discussing like she has this disease so she is not able to gain weight like that kind of things. And I do not like that, I feel like they are pitying or mocking me saying that I have sugar, consistently saying that I have sugar, and that makes me angry. (Laughs)

I: Have you experienced any such incidence where you were uncomfortable telling people that you have diabetes?

P: We used to hide it before, only a few of the family members knew then. Now it has normalized a little as we have learned about the disease .

I: (coughs) sorry and in times of covid, being a Type 1 diabetes person how you feel living in the time of Covid, please share your experience.

P: They say that sickly people have low immunity and they can get it easily. And that used to scare me, so I took care of myself. And sometimes when I used to get a fever I used to get scared. Once I had a fever and also symptoms similar to Covid so I went for the PCR test but the report was negative. Later it was found out that I was having low blood pressure so the symptoms were due to that cause. Sometimes my pressure and sugar are low so it becomes difficult.

I: Do you have experience of not going out or not going to college during this covid time?

P: No nothing like that, just my pressure became low, I just stayed in the shop and did not have time since one person cannot handle the store, but when it was low I did not stay in the shop for a few days.

I: ……., this has been a fun conversation, is there anything you want to share that you feel I did not ask you,……?

P: No nothing like that.

I: Thank you for giving your time, …..

P: Yes, I made you wait a long time last time as well.
